# The effect of secondary inorganic aerosols, soot and the geographical origin of air mass on acute myocardial infarction hospitalisations in Gothenburg, Sweden during 1985–2010: a case-crossover study

**DOI:** 10.1186/1476-069X-13-61

**Published:** 2014-07-29

**Authors:** Janine Wichmann, Karin Sjöberg, Lin Tang, Marie Haeger-Eugensson, Annika Rosengren, Eva M Andersson, Lars Barregard, Gerd Sallsten

**Affiliations:** 1 Occupational and Environmental Medicine, Sahlgrenska University Hospital and University of Gothenburg, Medicinaregatan 16A, Gothenburg, Sweden; 2 School of Health Systems and Public Health, University of Pretoria, Pretoria, South Africa; 3 IVL Swedish Environmental Research Institute, Gothenburg, Sweden; 4 Department of Emergency and Cardiovascular Medicine, Sahlgrenska University Hospital, Gothenburg, Sweden; 5 Department of Emergency and Cardiovascular Medicine, University of Gothenburg, Gothenburg, Sweden

**Keywords:** Air pollution, Secondary inorganic aerosols, Soot, Geographical air mass origin, Acute myocardial infarction, Hospitalisations, Gothenburg, Case-crossover

## Abstract

**Background:**

The relative importance of different sources of air pollution for cardiovascular disease is unclear. The aims were to compare the associations between acute myocardial infarction (AMI) hospitalisations in Gothenburg, Sweden and 1) the long-range transported (LRT) particle fraction, 2) the remaining particle fraction, 3) geographical air mass origin, and 4) influence of local dispersion during 1985–2010.

**Methods:**

A case-crossover design was applied using lag0 (the exposure the same day as hospitalisation), lag1 (exposure one day prior hospitalisation) and 2-day cumulative average exposure (CA2) (mean of lag0 and lag1). The LRT fractions included PM_ion_ (sum of sulphate, nitrate and ammonium) and soot measured at a rural site. The difference between urban PM_10_ (particulate matter with an aerodynamic diameter smaller than 10 μm) and rural PM_ion_ was a proxy for locally generated PM_10_ (PM_rest_). The daily geographical origin of air mass was estimated as well as days with limited or effective local dispersion. The entire year was considered, as well as warm and cold periods, and different time periods.

**Results:**

In total 28 215 AMI hospitalisations occurred during 26 years. PM_10_, PM_ion_, PM_rest_ and soot did not influence AMI for the entire year. In the cold period, the association was somewhat stronger for PM_rest_ than for urban PM_10;_ the strongest associations were observed during 1990–2000 between AMI and CA2 of PM_rest_ (6.6% per inter-quartile range (IQR), 95% confidence interval 2.1 to 11.4%) and PM_10_ (4.1%, 95% CI 0.2% − 8.2%). Regarding the geographical air mass origins there were few associations. Days with limited local dispersion showed an association with AMI in the cold period of 2001–2010 (6.7%, 95% CI 0.0% − 13.0%).

**Conclusions:**

In the cold period, locally generated PM and days with limited local dispersion affected AMI hospitalisations, indicating importance of local emissions from e.g. traffic.

## Background

Cardiovascular disease (CVD) is the number one cause of death globally and also in European countries, such as Sweden [1]. Ischemic heart disease (IHD) is responsible for 61% of the 2 million CVD deaths in Europe annually, and stroke accounts for most of the rest [1]. Acute myocardial infarction (AMI) is the most important manifestation of ischemic heart disease.

The major risk factors for CVD are age, sex, smoking, low physical activity, increased waist circumference, diabetes and hypertension [2,3]. Evidence is increasing on the effects of non-traditional risk factors, such as air pollution and weather (e.g. temperature) on CVD mortality and morbidity, specifically as short-term risk factors [4-6]. Nevertheless, many research gaps remain [7].

Numerous short-term epidemiological studies as well as in-vivo and in-vitro toxicological studies reported on the toxicity of the physical and chemical properties of particulate matter, but effects seem not to be limited to single properties or sources [4,8,9].

Polluted air may be local in origin or transported over long distances and the composition may vary [10]. The composition of air masses from Eastern Europe, where coal heating continues to dominate, differs from that of Western Europe [11]. Emissions have also dramatically changed over the past 20–30 years, as in the case of sulphur dioxide. The geographical origin of the air mass can be traced through a combination of measurements and calculations using meteorological models (trajectory analysis) [12]. Large differences in concentrations may occur; e.g. with respect to trace elements and particulate matter with an aerodynamic diameter smaller than 2.5 μm (PM_2.5_) and black smoke [13-15]. Transition metals and polyaromatic hydrocarbons are possible causes of oxidative stress and inflammation [16]. Trajectory analyses have been used only sporadically for studies on health effects of air pollutants and not previously in a case-crossover design [17].

Another research gap is that few epidemiological studies focused on subtypes of CVD, such as AMI hospitalisations [4-6]. It is assumed that the pathophysiology of subtypes of CVD is different and therefore different associations between air pollution and these subtypes can be anticipated.

The REVIHAAP report concluded that there is currently no threshold level (i.e. no safe level) for PM_10_, PM_2.5_, nitrogen dioxide (NO_2_) and ground-level ozone (O_3_) and that the concentration-response functions are mostly linear [7,18]. Hence it is appropriate to conduct studies in cities with ‘relatively low levels’ like Gothenburg.

The objective of this study was to investigate whether there is an association between AMI hospitalisations in Gothenburg, Sweden and surrogates for long-range transported (LRT) air pollutants (ions and soot) and the more locally emitted parts of PM_10_. Another aim was to determine the association between AMI hospitalisations and geographical air mass origin (i.e. a surrogate for LRT air pollution and its composition). Gothenburg is a suitable area for such studies, since this region is characterised by a rather large meteorological variability resulting in air mass transport both from clean and polluted areas [14,15,19]. A case-crossover study design was applied to the study period of 26 years (1985–2010).

## Methods

### Population and register data

Sweden has a publicly financed health care system with hospital care available to all citizens at low cost. Swedish hospitals register principal and contributory discharge diagnoses for all patients in the national hospital discharge register. In the present study we used data from this register with coverage of all hospitalisations in Gothenburg since 1970.

The study population includes all cases of AMI events registered in Gothenburg from 1 January 1985 to 31 December 2010. The study period was determined by the availability of air pollution data. The International Classification of Diseases (ICD) versions 8 and 9 (ICD-8, ICD-9) were used from 1985 to 1996 and version 10 (ICD 10) from 1997 and onwards.

Hospitalisation for an AMI was defined as a discharge (dead or alive) with a principal diagnosis of ICD 8–9 410 (until 1996) or ICD 10 I21. Only emergency hospitalisations to any of the five hospitals within Gothenburg (1.7 to 5.8 km from the urban background monitoring station) were included. AMI hospitalisations that occurred within 28 days after a previous AMI hospitalisation were excluded (3 068 admissions) as readmissions following discharge for AMI are quite high [20]. Due to the design of the case-crossover study, overlap between being a case and a control may occur for these 3 068 admissions. For information regarding the case-crossover study, see s*tatistical analysis* section.

### Ethics

Registry based health outcome data were applied in this study and all identifying variables were deleted. The study was approved by the institutional review board of the University of Gothenburg which waived the need for written informed consent.

### Long-range transported air pollution data

*PM*_*ion*_*, sulphate, nitrate and ammonium ions and soot*

PM_ion_ (estimated sum of sulphate (SO_4_^2−^), nitrate (NO_3_^−^) and ammonium (NH_4_^+^) ions) and the three individual ions were used as surrogates for LRT secondary PM [21].

$$\begin{array}{l} PM_{\mathrm{ion}}=3.0{\left[SO_{4}^{2-}\right]}_{s}+4.4{\left[NO_{3}^{-}+\mathrm{HN}O_{3}\right]}_{N}\\ \;+1.3{\left[NH_{4}^{+}+NH_{3}\right]}_{N} \end{array}$$

where the subscripts S and N denote that the mass has been given as the equivalent mass of sulphur or nitrogen, respectively. In order to treat the particulate concentration variables in a comparable manner, these values were converted to equivalent masses of the SO_4_^2−^, NO_3_^−^ and NH_4_^+^ ions, using the conversion factors 3.0, 4.4 and 1.3, respectively [21]. The three ions were measured by IVL Swedish Environmental Research Institute on behalf of the Swedish Environmental Protection Agency, within the national air quality monitoring network at a rural background site located in Råö, 45 km south of the city centre [19,22]. At the same place soot was monitored by a reflectance method [23].

### Geographical origin of air masses

The geographical origins of the air masses that pass Gothenburg were applied as another surrogate for the LRT fraction of air pollution and its composition. For all 9 496 days two-dimensional trajectories were calculated and further analysed, using the HYSPLIT model to show the geographical origin of the air masses for these days.

The trajectories were divided into six classes which define the geographical origin of the air mass (Southern Scandinavia, Northern Scandinavia, Baltic Sea, Eastern Europe, United Kingdom (UK)/Denmark (DK)/North Sea and North Atlantic, Additional file 1), based on large scale daily synoptic weather data from NCEP/NCAR (National Centre for Environmental Prediction/National Centre for Atmospheric Research [19,24,25].

Days were also classified as either limited local dispersion days or effective local dispersion days. Limited local dispersion day means a day with inversion and/or stable air stratification suppressing the dispersion of locally emitted air pollutants. On the limited local dispersion days the concentration contribution from LRT is almost unchanged. However, the local contribution will be higher. We applied the Lambs weather type classification [24], along with the concentration of PM_ion_ as a surrogate for LRT [19] and nitrogen oxides (NO_x_) as a surrogate for limited urban dispersion [25] for the classification.

### Meteorological and urban air pollution data

Temperature, relative humidity (HMP45a probe), PM_10_ and PM_2.5_ (tapered element oscillating microbalance instrument) were measured at the urban background monitoring station by the Environment Office in Gothenburg. NO_2_, NO_x_ and O_3_ were also measured at the same station [26].

Daily averages were applied in the statistical analyses. Missing values were not imputed. For 437 days the PM_10_ levels were lower than 5 μg.m^−3^ and were set as 5 μg.m^−3^. The difference between the urban PM_10_ and the rural PM_ion_ levels was used as a surrogate for the more locally generated PM fraction (hereafter PM_rest_). PM_10_ and PM_2.5_ data are available only from 1990 and 2006, respectively.

### Statistical analysis

The time-stratified case-crossover design was applied to investigate the association between AMI hospitalisations and PM_10_, PM_2.5_, surrogates for LRT air pollutants (PM_ion_ fraction, three individual ions, soot, geographical origin of the air masses), PM_rest_ fraction and limited/effective local dispersion. The case-crossover design was developed as a variant of the case–control design to study the effects of transient exposures on emergency events, comparing each person’s exposure in a time period just prior to a case-defining event with the person’s exposure at other times [27]. A time-stratified approach was applied to select the control days, defining the day of hospitalisation as the case day and the same day of the other weeks in the same month and year as control days. With this approach even very strong confounding of exposure by seasonal patterns is controlled by design [28-30]. The data were analysed using conditional logistic regression analysis (PROC PHREG in SAS 9.2, SAS Institute, Cary, NC).

Lag0 (the exposure the same day as hospitalisation) and lag1 (exposure one day prior hospitalisation) were investigated, as well as the 2-day cumulative average (CA2, mean of lag0-1). The values of CA2 were set as missing if any of the values of lag0 or lag1 were missing. Control days for lag1 and CA2 were defined as for lag0.

All models were adjusted for public holidays (as a binary variable), temperature and relative humidity (same lag as PM_10_, PM_ion_, PM_rest_, the three individual ions, PM_2.5_, soot, geographical origin of air mass and limited/effective local dispersion). PM_10_, PM_ion_, PM_rest_, the three individual ions, PM_2.5_, soot and meteorological variables were included as linear terms in the models. Previous studies reported a linear relationship between PM_10_ and AMI hospitalisations [31-34]. The linearity of the relationship between AMI hospitalisations and the meteorological variables (temperature and relative humidity) was confirmed in another study [26].

Models that included the geographical origin of the air mass were run for lag0 and lag1, but not for CA2, as the geographical origin of the air mass is a nominal variable. Southern Scandinavia was selected as the reference category as this is where Gothenburg is located, although such air masses could also come from adjacent countries (Additional file 1) [19]. Models that included limited/effective local dispersion days were run in the same way.

Although individual factors (e.g. sex, age, season) cannot be examined due to the nature of the case-crossover design where each person is his/her own control, the susceptibility to a pollutant may differ between subgroups (sex, age, season). Warm and cold months have different temperatures, which many lead to different time-activity behaviour and also a difference in the composition of the air pollution mix due to fewer emissions from heating. Hence, association was investigated in stratified analyses, where the effect of the pollutant was compared between the spring/summer half-year (April − September) (hereafter warm period) and the autumn/winter half-year (October − March) (hereafter cold period). This approach was applied in previous studies [35-37].

A further stratification was made into time periods by 1985(1990) − 2000 and 2001–2010. Data were available from 1985 for soot, ions and geographical air mass origins as well as for days with limited or effective local dispersion. As mentioned before PM_10_ data were only available from 1990. A reason for this stratification is that air pollution emissions changed in Europe during the last two decades, with large reductions in SO_x_, NO_x_ and NH_x_ emissions during the late 1990s [38]. The emission changes might have influenced the composition and toxicology of air pollution, such as PM, in the atmosphere. The earlier time period (1985/1990 − 2000) cannot be investigated for PM_2.5_ therefore we did not investigate this second stratification for PM_2.5_. As mentioned before PM_2.5_ data were only available from 2006. To test for different effects of the pollutants in different subgroups, models with interaction terms were applied.

For the PM_10_, PM_ion_, PM_rest_, the three individual ions, PM_2.5_ and soot, odds ratios (OR) and the 95% confidence intervals (CI) were calculated per inter-quartile range (IQR) increase, which provide the magnitude-of-risk estimates that are comparable across the exposure variables. The results are presented as the per cent excess risk in AMI hospitalisations per IQR increase in the air pollution levels using the following calculation: (exp^(βxIQR)^ – 1) × 100%, where β is the model estimate. For analysis of a given lagged exposure, a case was automatically dropped if exposure data were not available for the case and at least one control day.

## Results

Table 1 indicates the characteristics of the 28 215 AMI hospitalisations (of 22 475 people) during the study period. The mean age for AMI hospitalisations was 74 years. More hospitalisations occurred among men, people older than 75 years and during the cold period. Additional file 2 illustrates the time-series of the daily number of AMI hospitalisations during the study period. The number of AMI hospitalisations varied from 0 to 23 per day.

**Table 1:** Characteristics of the acute myocardial infarction hospitalisation in Gothenburg, Sweden during 1 January 1985–31 December 2010

	**No. cases**	**%**
**Total**	28215	100.0
**Sex**		
Male	16627	58.9
Female	11588	41.1
**Age (years)**		
23 − 35	62	0.2
36 − 55	2459	8.7
56 − 65	3951	14.0
66 − 75	7390	26.2
76 − 84	9122	32.3
85 − 102	5231	18.5
**Seasonal period**		
Warm	13381	47.4
Cold	14834	52.6
**Time period**		
1985 − 2000	18264	64.7
1990 − 2000	12109	42.9
2001 − 2010	9951	35.3

Warm period: April − September, cold period: October − March.

Time period: Air pollution emissions in Europe changed during the last two decades, with large reductions in SO_x_, NO_x_ and NH_x_ emissions during the late 1990s [38]. PM_10_ and PM_rest_ available from 1990–2010.

Table 2 provides an overview of the daily levels of PM_10_, PM_ion_, PM_rest_, the three individual ions, PM_2.5_, soot, temperature and relative humidity. The daily WHO and EU air quality limits (50 μg.m^−3^) for the PM_10_ levels were exceeded on 60 days at the urban background site during the 21-year study period (Additional file 3) [8].

**Table 2:** Descriptive statistics for daily meteorological and air pollutant levels (lag0) in Gothenburg, Sweden (1 January 1985–31 December 2010)

					**Percentiles**			
	**No. days missing data**	**Mean**	**SD**	**Range**	**25**^**th**^	**50**^**th**^	**75**^**th**^	**IQR**
**All year (9496 days)**								
Temperature (°C)	146	8.5	7.2	−22.0 − 26.2	3.3	8.4	14.4	11.1
Relative humidity (%)	637	77.4	12.5	15.0 − 99.9	69.8	79.4	87.1	17.3
PM_10_ (μg.m^−3^)	2004	15.9	9.2	5 − 78.1	9.6	14.0	20.0	10.4
PM_ion_ (μg.m^−3^)	899	6.6	5.6	0.4 − 63.6	3.0	5.0	8.4	5.4
PM_rest_ (μg.m^−3^)	2373	10.0	8.0	0.0 − 70.2	4.2	8.2	13.2	9.0
SO_4_^2−^ (μg.m^−3^)	262	1.0	1.0	0.0 − 18.5	0.4	0.7	1.2	0.8
Total NO_3_^−^ (μg.m^−3^)	746	0.6	0.7	0.0 − 12.6	0.2	0.4	0.8	0.6
Total NH_4_^+^ (μg.m^−3^)	696	1.0	1.0	0.0 − 12.3	0.4	0.7	1.3	0.9
PM_2.5_ (μg.m^−3^)	7718	7.7	4.5	0.6 − 40.9	4.7	6.7	9.3	4.6
Soot (μg.m^−3^)	319	2.6	3.6	0.8 − 48.2	0.8	1.2	2.9	1.7
**Warm period (4758 days)**								
Temperature (°C)	47	13.8	4.6	−1.1 − 26.2	10.9	14.3	16.9	6.1
Relative humidity (%)	304	72.2	12.3	32.0 − 99.0	64.7	73.5	81.2	16.5
PM_10_ (μg.m^−3^)	973	15.4	8.7	5 − 76.0	9.5	13.5	19.0	9.5
PM_ion_ (μg.m^−3^)	433	6.5	5.1	0.5 − 63.6	3.2	5.0	8.3	5.1
PM_rest_ (μg.m^−3^)	1106	9.5	7.3	0.0 − 65.0	4.3	7.8	12.4	8.0
SO_4_^2−^ (μg.m^−3^)	126	1.0	0.9	0.0 − 13.3	0.4	0.7	1.2	0.8
Total NO_3_^−^ (μg.m^−3^)	347	0.6	0.7	0.0 − 9.9	0.2	0.4	0.7	0.5
Total NH_4_^+^ (μg.m^−3^)	333	1.1	0.9	0.0 − 9.6	0.5	0.8	1.3	0.8
PM_2.5_ (μg.m^−3^)	3845	7.2	4.2	0.9 − 35.6	4.5	6.3	8.6	4.1
Soot (μg.m^−3^)	168	1.7	2.1	0.8 − 23.7	0.8	0.8	1.9	1.1
**Cold period (4738 days)**								
Temperature (°C)	99	3.1	5.0	−22.0 − 16.3	0.2	3.5	6.4	6.2
Relative humidity (%)	333	82.7	10.3	15.0 − 99.9	77.4	84.8	90.1	12.7
PM_10_ (μg.m^−3^)	1031	16.6	9.7	5 − 78.1	9.7	14.6	20.8	11.1
PM_ion_ (μg.m^−3^)	466	6.7	6.0	0.4 − 63.6	2.8	5.0	8.6	5.8
PM_rest_ (μg.m^−3^)	1267	10.6	8.6	0.0 − 70.2	4.0	8.6	14.1	10.1
SO_4_^2−^ (μg.m^−3^)	136	1.1	1.1	0.0 − 18.5	0.4	0.7	1.3	0.9
Total NO_3_^−^ (μg.m^−3^)	399	0.6	0.7	0.0 − 12.6	0.2	0.4	0.8	0.6
Total NH_4_^+^ (μg.m^−3^)	363	1.0	1.1	0.0 − 12.3	0.3	0.6	1.3	1.0
PM_2.5_ (μg.m^−3^)	3873	8.2	4.8	0.6 − 40.9	5.0	7.0	10.0	5.0
Soot (μg.m^−3^)	151	3.5	4.5	0.8 − 48.2	0.8	2.0	4.2	2.2

SD: Standard deviation, IQR: Interquartile range.

Warm period: April − September, cold period: October − March.

PM_10_ and PM_rest_ available from 1990–2010 and PM_2.5_ available from 2006–2010.

Additional files 4 and 5 illustrate the PM_10_, PM_ion_, PM_rest_, the three individual ions, PM_2.5_, soot, temperature and relative humidity and additionally the NO_x_, NO_2_, O_3_ and wind speed levels by the six geographical air mass origins. Descriptive results of NO_x_, NO_2_ and O_3_ were reported in another publication [26]. Compared to Southern Scandinavia, NO_2_ and NO_x_ levels were higher when the air mass originated from Northern Scandinavia. In contrast, PM_10_, PM_ion_, the three individual ions, PM_2.5_, soot, temperature and relative humidity were lower. Higher PM_10_, PM_ion_, sulphate ion, ammonium ion, PM_2.5_, soot and relative humidity but lower NO_2_ and NO_x_ levels were observed when the air mass origin was from Eastern Europe compared to Southern Scandinavia. The geographical air mass origin variable is thus not just a surrogate for LRT air pollution and its composition, but a combination of both the large-scale meteorological conditions as well as the large-scale air pollution transportation pattern.

Table 3 displays the Spearman correlations between PM_10_, PM_ion_, PM_rest_, the three individual ions, PM_2.5_, soot, temperature and relative humidity during the entire year, and the warm and cold periods. Relative humidity was in general inversely correlated with the other exposure variables, except with the three individual ions and PM_ion_. Temperature had a weak positive correlation with the other exposure variables, except with PM_rest_ in the entire year and the cold period. PM_10_ had a positive and stronger correlation with PM_rest_ and PM_2.5_ than with PM_ion_. PM_ion_ had a stronger correlation with PM_2.5_ than with PM_10_. PM_ion_ had a weak inverse correlation with PM_rest_ in the entire year and the cold period. The three individual ions were strongly correlated. Relatively high correlations were found between soot and sulphate and nitrate ions.

**Table 3:** Spearman correlation coefficients between exposure variables (daily lag0) in Gothenburg, Sweden during the entire year, warm and cold periods (1 January 1985–31 December 2010)

**Entire year**	**Temp**	**PM**_**10**_	**PM**_**ion**_	**PM**_**rest**_	**SO**_**4**_^**2−**^	**Total NO**_**3**_^**−1**^	**Total NH**_**4**_^**+**^	**PM**_**2.5**_	**Soot**
**Rel. hum**	−0.331	−0.127	0.217	−0.272	0.208	0.280	0.134	0.115	0.235
	8859^a^	7445	8139	7089	8622	8255	8311	1778	8588
**Temp**		0.003^b^	0.130	−0.033	0.082	0.115	0.179	−0.026	−298
		7482	8473	7115	9097	8619	8667	1778^b^	9038
**PM**_**10**_			0.459	0.803	0.346	0.321	0.338	0.567	0.284
			7123	7123	7281	7212	7247	1773	7268
**PM**_**ion**_				−0.059	0.877	0.833	0.862	0.615	0.535
				7123	8597	8597	8597	1744	8411
**PM**_**rest**_					−0.111	−0.144	−0.129	0.289	0.022^b^
					7123	7123	7123	1739	6976
**SO**_**4**_^**2−**^						0.625	0.770	0.613	0.569
						8677	8724	1749	9025
**Total NO**_**3**_^**−1**^							0.761	0.500	0.377
							8669	1747	8509
**Total NH**_**4**_^**+**^								0.586	0.540
								1747	8564
**PM**_**2.5**_									0.479
									1752
**Warm period**	**Temp**	**PM**_**10**_	**PM**_**ion**_	**PM**_**rest**_	**SO**_**4**_^**2−**^	**Total NO**_**3**_^**−1**^	**Total NH**_**4**_^**+**^	**PM**_**2.5**_	**Soot**
**Rel. hum**	−0.161	−0.157	0.169	−0.306	0.189	0.219	0.104	0.005^b^	0.019^b^
	4454	3781	4139	3648	4342	4206	4214	913	4305
**Temp**		0.109	0.148	0.088	0.167	0.090	0.135	0.191	−0.057
		3781	4290	3648	4589	4372	4384	913	4546
**PM**_**10**_			0.547	0.805	0.423	0.405	0.471	0.620	0.339
			3652	3652	3692	3694	3697	911	3673
**PM**_**ion**_				0.033	0.887	0.857	0.871	0.697	0.502
				3652	4325	4325	4325	896	4219
**PM**_**rest**_					−0.030^b^	−0.059	0.012	0.323	0.124
					3652	3652	3652	894	3568
**SO**_**4**_^**2−**^						0.665	0.788	0.657	0.548
						4362	4374	899	4514
**Total NO**_**3**_^**−1**^							0.745	0.611	0.325
							4373	898	4271
**Total NH**_**4**_^**+**^								0.696	0.551
								897	4286
**PM**_**2.5**_									0.424
									895
**Cold period**	**Temp**	**PM**_**10**_	**PM**_**ion**_	**PM**_**rest**_	**SO**_**4**_^**2−**^	**Total NO**_**3**_^**−1**^	**Total NH**_**4**_^**+**^	**PM**_**2.5**_	**Soot**
**Rel. hum**	0.183	−0.170	0.325	−0.332	0.239	0.410	0.298	0.142	0.158
	4405^a^	3664	4000	3441	4280	4049	4097	865	4283
**Temp**		0.054	0.215	−0.068	0.138	0.262	0.140	0.011^b^	−0.120
		3701	4183	3467	4508	4247	4283	865	4492
**Warm period**	**Temp**	**PM**_**10**_	**PM**_**ion**_	**PM**_**rest**_	**SO**_**4**_^**2−**^	**Total NO**_**3**_^**−1**^	**Total NH**_**4**_^**+**^	**PM**_**2.5**_	**Soot**
**PM**_**10**_			0.381	0.801	0.275	0.248	0.239	0.516	0.238
			3471	3471	3589	3518	3550	862	3595
**PM**_**ion**_				−0.140	0.869	0.815	0.864	0.567	0.630
				3471	4272	4272	4272	848	4192
**PM**_**rest**_					−0.187	−0.218	−0.240	0.251	−0.084
					3471	3471	3471	845	3408
**SO**_**4**_^**2−**^						0.592	0.769	0.581	0.644
						4315	4350	850	4511
**Total NO**_**3**_^**−1**^							0.784	0.429	0.458
							4296	849	4238
**Total NH**_**4**_^**+**^								0.564	0.664
								850	4278
**PM**_**2.5**_									0.518
									857

^a^Number of days less than 9496 in the entire year, 4758 in the warm and 4738 in the cold period due to missing exposure data.

^b^p-value ≥ 0.05, otherwise p-value > 0.05.

Warm period: April − September, cold period: October − March.

PM_10_ and PM_rest_ available from 1990–2010 and PM_2.5_ available from 2006–2010.

**Figure 1:**
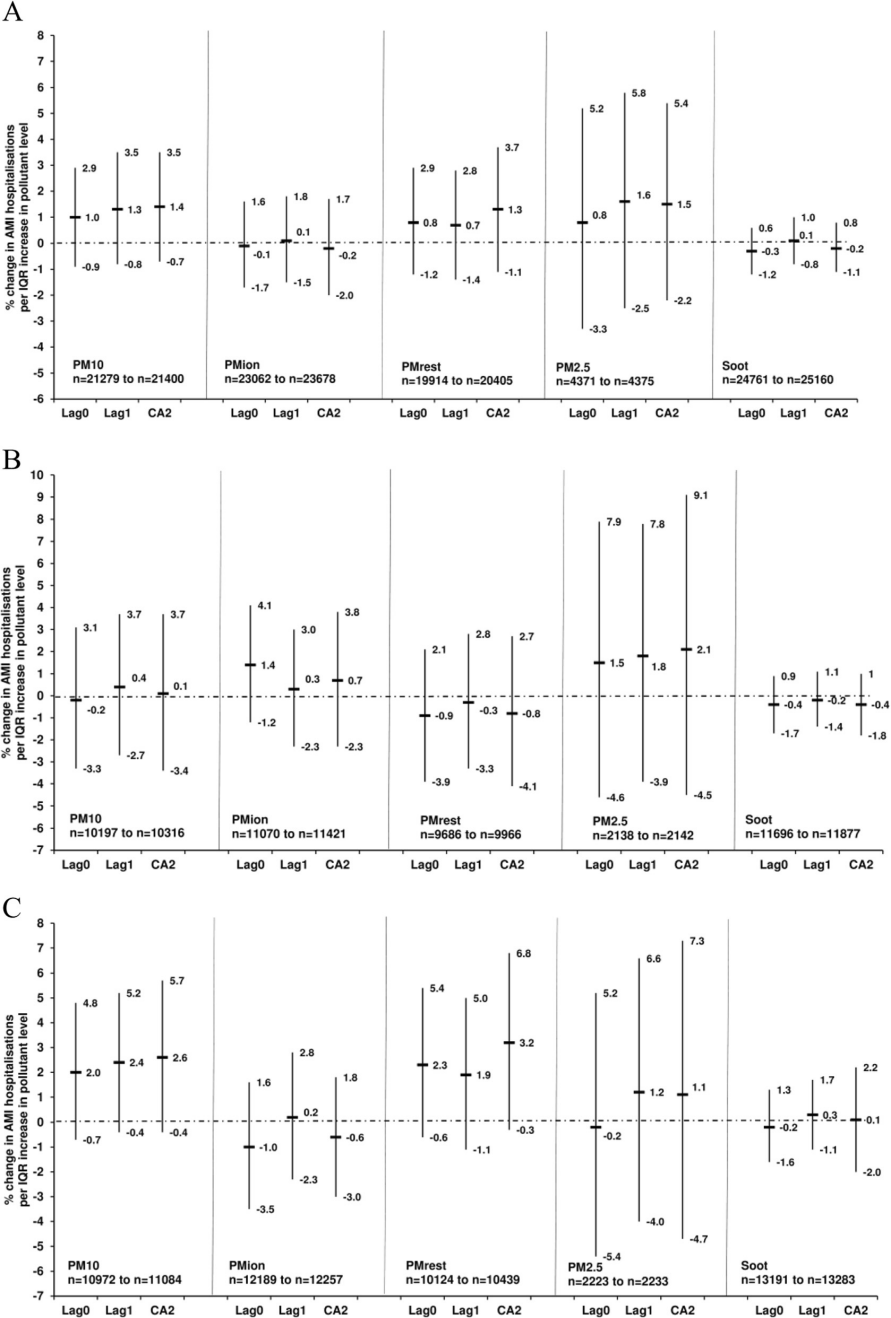
**Association between the lag0, lag1 and the 2-day cumulative average of PM**_**10**_**, PM**_**ion**_**, PM**_**rest**_**, PM**_**2.5**_, soot and acute myocardial infarction hospitalisations in Gothenburg, Sweden (1985/1990*/2006*) − 2010) as percentage change in risk (%) and 95% confidence intervals during (A) the entire year, (B) warm period (April − September) and (C) cold period (October − March). Models adjusted for temperature, relative humidity and public holidays. Number of cases (n) used in the models is less than the original number due to missing exposure data. *PM_10_ and PM_rest_ data from 1990 and PM2.5 data from 2006.

**Figure 2:**
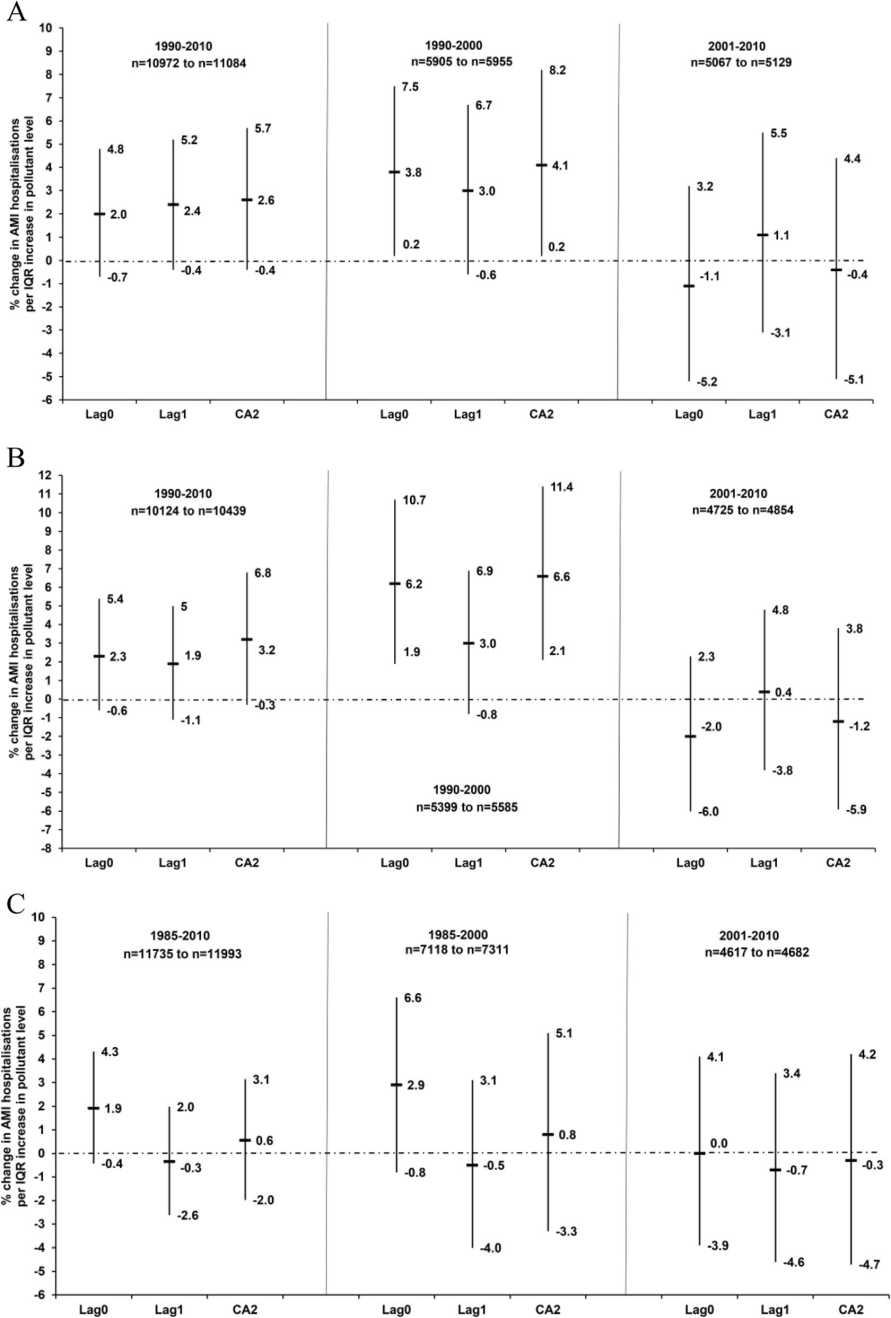
**Association between the lag0, lag1 and the 2-day cumulative average of (A) PM**_**10 **_**in the cold period (October − March), (B) PM**_**rest **_in the cold period (October − March) and (C) sulphate ion in the warm period (April − September) and acute myocardial infarction hospitalisations in Gothenburg, Sweden (1985(1990*) − 2010) as percentage change in risk (%) and 95% confidence intervals. Models adjusted for temperature, relative humidity and public holidays. Number of cases (n) used in the models is less than the original number due to missing exposure data. *PM_10_ and PM_rest_ data from 1990.

Figure 1 illustrates the% change in the AMI hospitalisations per IQR increase in the lags of PM_10_, PM_ion_, PM_rest_, PM_2.5_ and soot during the entire year, warm and cold periods. The point estimates for PM_10_ (CA2) and PM_2.5_ (CA2) for the entire year were similar, 1.4% and 1.5% per IQR, but the 95% CI included zero. The 95% CI intervals of PM_2.5_ are wider due to the shorter study period (2006–2010). The point estimate for the increase in AMI in the cold period was somewhat larger for PM_rest_ (3.2%, 95% CI −0.3% − 6.8%) than for PM_10_ (2.6%, 95% CI −0.4% − 5.7%). When the cold period was restricted to 1990–2000, stronger positive associations were observed between AMI hospitalisations and PM_rest_ (6.6%, 95% CI 2.1% − 11.4%) and PM_10_ (4.1%, 95% CI 0.2% − 8.2%) (Figure 2).

Although there was no association between AMI and PM_ion_, a positive association with sulphate ion was observed in the warm period (1.9%, 95% CI −0.4% − 4.3%) (Figure 2, Additional file 6), which was stronger when the warm period was restricted to 1985–2000 (Figure 2). In the latter restricted period, the sample sizes were however much smaller than those in Figure 1. Caution should however be applied to the stratified results as the interaction terms were not statistically significant.

**Figure 3:**
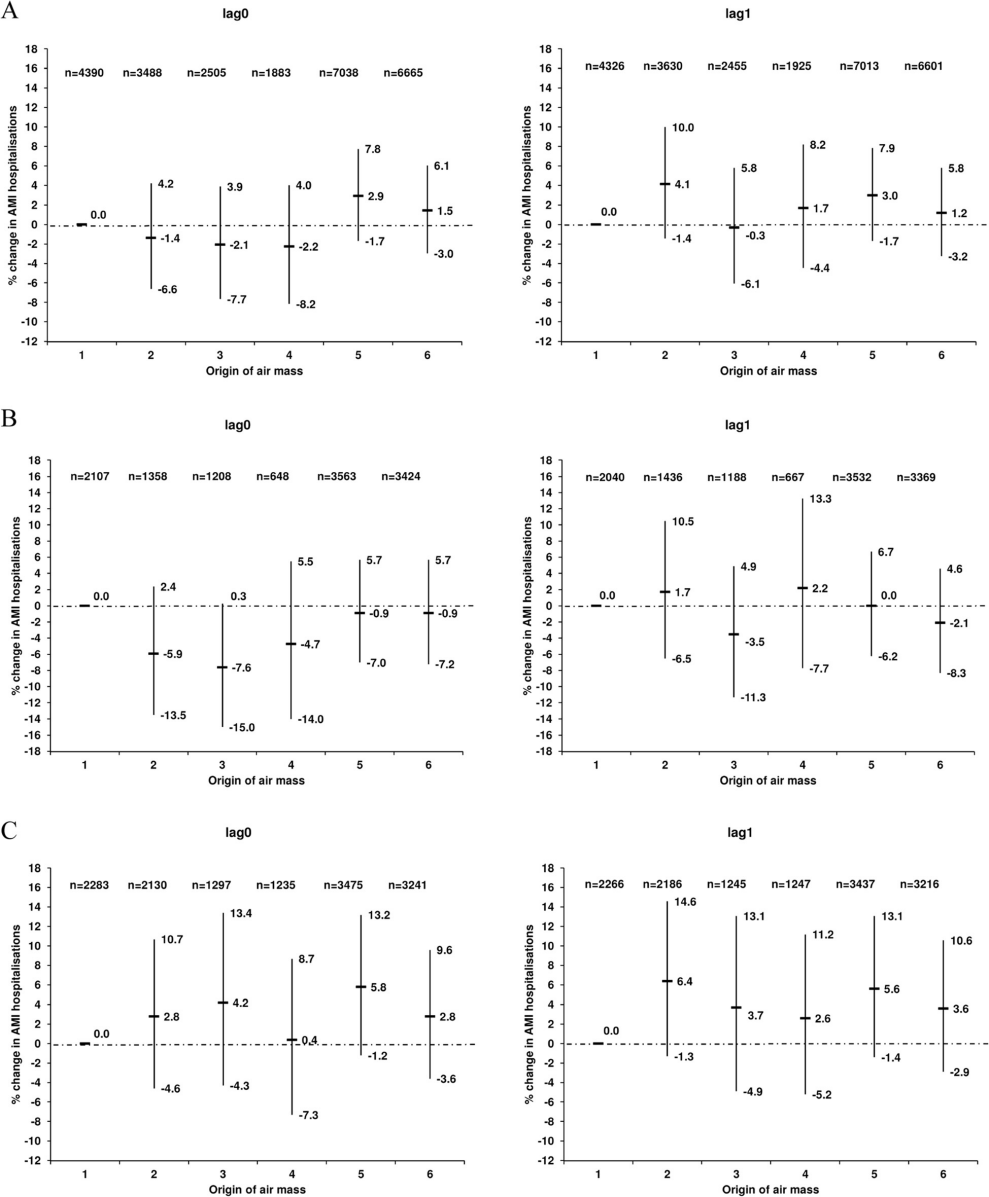
**Association between the lag0 and lag1 of the geographical origin of the air masses and acute myocardial infarction hospitalisations in Gothenburg, Sweden (1985–2010) as percentage change in risk (%) and 95% confidence intervals during the (A) entire year, (B) warm period (April − September) and (C) cold period (October − March).** Models adjusted for temperature, relative humidity and public holidays. Number of cases (n) used in the models is less than the original number due to missing exposure data. Geographical origin of air masses: 1: Southern Scandinavia, 2. Northern Scandinavia, 3: Baltic Sea, 4: Eastern Europe, 5. UK/DK/North Sea and 6: North Atlantic. The reference category is 1: Southern Scandinavia.

Figure 3 indicates the% change in the AMI hospitalisations for the five geographical air mass origins relative to that of Southern Scandinavia during the entire year, warm and cold periods. There was no clear association between AMI hospitalisations and lag0 of the geographical air mass origin when the entire year was considered. In the unadjusted model, a 5.1% (95% CI 0.1% − 10.3%) increase in AMI was observed when the air mass originated the day before admission (lag1) in Northern Scandinavia (relative to Southern Scandinavia) (Additional file 7). Adjusting further for temperature, relative humidity and public holidays resulted in a weaker association (4.1%, 95% CI −1.4% − 10.0%, entire year, lag1), but with somewhat higher point estimates in the cold period (Figure 3). When the cold period was analysed separately for different time periods, stronger positive associations were observed for 2001–2010 when the air mass originated in Northern Scandinavia (12.6% increase in AMI for lag1), UK/DK/North Sea (16.0% for lag0) and North Atlantic (13.4% significant increase at lag0, 15.1% at lag1) relative to Southern Scandinavia (Additional file 8). The sample sizes were however much smaller than those in Figure 3. Again, caution should be applied to the stratified results as the interaction terms were not significant.

Additional file 9 illustrates the PM_10_ and PM_rest_ levels by the six geographical air mass origins, after stratification by the entire year, warm and cold periods and also by the time periods 1990–2010, 1990–2000, and 2001–2010. The PM_10_ and PM_rest_ levels were highest during the 2001–2010 period (Additional file 10).

**Figure 4:**
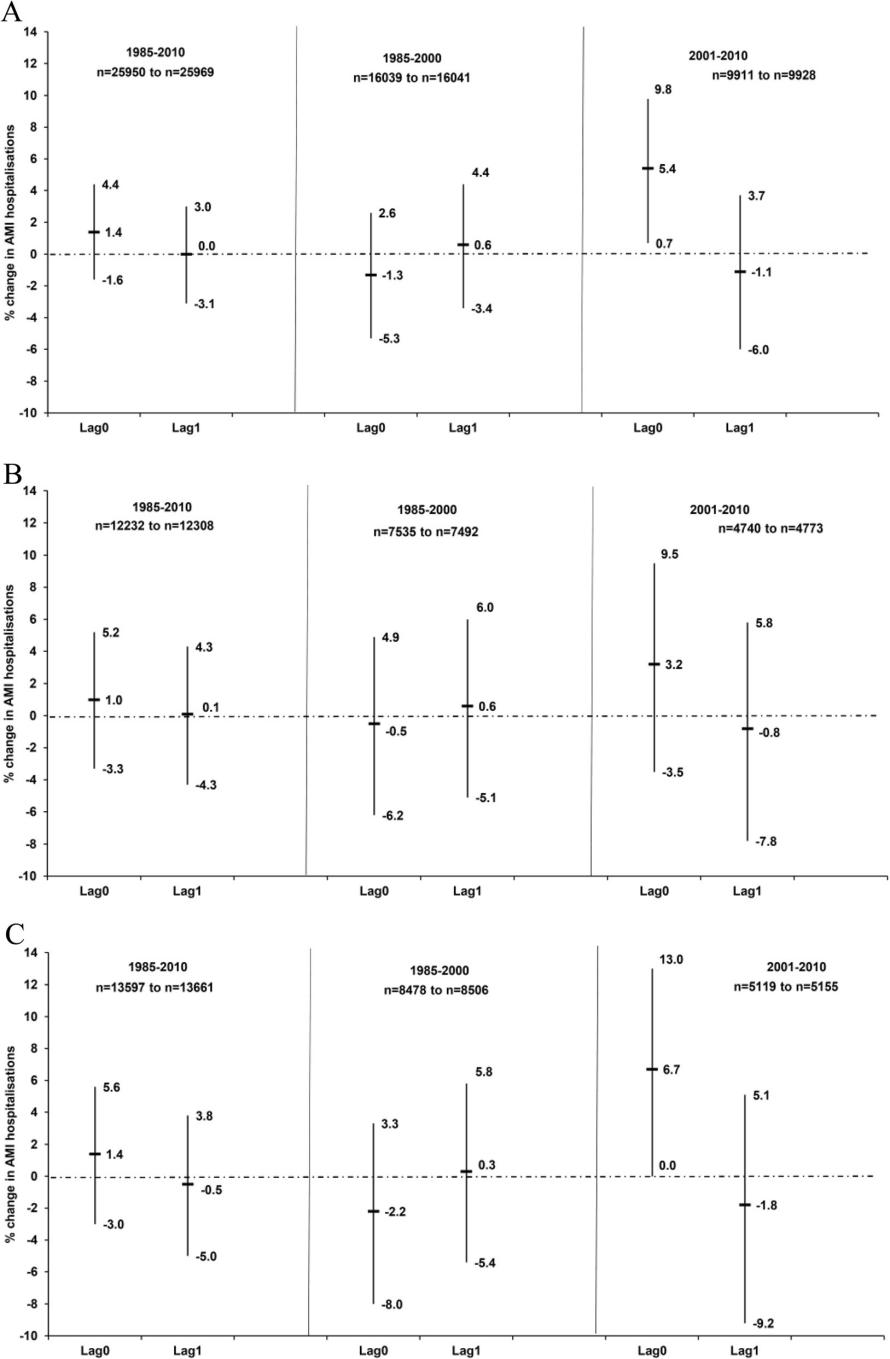
**Association between the lag0 and lag1 of days with limited local dispersion compared to effective local dispersion and acute myocardial infarction hospitalisations in Gothenburg, Sweden (1985–2010) as percentage change in risk (%) and 95% confidence intervals during the (A) entire year, (B) warm period (April − September) and (C) cold period (October − March).** Models adjusted for temperature, relative humidity and public holidays. Number of cases (n) used in the models is less than the original number due to missing temperature and relative humidity data.

Finally we examined the influence of air pollution dispersion. Days with limited local dispersion had a strong association at lag0 with AMI hospitalisations during 2001–2010 in the entire year (5.4%, 95% CI 0.7% − 9.8%), but not for lag1 (Figure 4). Caution should be applied to the stratified results as the interaction terms were not significant. For the entire year, warm and cold periods, days with local limited dispersion were much more common when the air masses originated from Eastern Europe, UK/DK/North sea and North Atlantic (<60% of the days) (Table 4).

**Table 4:** Distribution of days with limited/effective local dispersion in Gothenburg, Sweden by the six geographical air mass origins (1 January 1985–31 December 2010: 9496 days)

	**Entire year**		**Warm period: April − September**		**Cold period: October − March**	
	**Limited dispersion**	**Effective dispersion**	**Limited dispersion**	**Effective dispersion**	**Limited dispersion**	**Effective dispersion**
**Southern Scandinavia**	637	971	284	520	353	451
**Northern Scandinavia**	254	980	153	375	101	605
**Baltic Sea**	408	493	191	271	217	222
**Eastern Europe**	570	123	209	45	361	78
**UK/DK/North Sea**	2378	223	1189	202	1189	21
**North Atlantic**	1634	825	850	469	784	356
**Total**	5881	3615	2876	1882	3005	1733

## Discussion

We set out to address some of the research gaps on the association between CVD and air pollution [5,7,34]. First, we investigated whether there is an association between air pollution and AMI emergency room hospitalisations (a specific subtype of CVD). Secondly, we considered surrogates for LRT air pollutants (soot, ammonium, nitrate and sulphate ions), more locally emitted PM_10_ (PM_rest_), days with limited dispersion as well as the geographical origins of air masses that pass Gothenburg. Thirdly, the entire year as well as the warm (April − September) and cold periods (October − March) were investigated. Fourthly, we considered different time periods 1985(1990) − 2000, 2001–2010 and 1985(1990) − 2010.

We observed conflicting results with regards to seasonal and time periods, but found that locally generated PM was more important for AMI hospitalisations.

An IQR (10 μg.m^−3^) increase in lag0 of PM_10_ was associated with an insignificant increase in AMI hospitalisation (1%) when the entire 26-year period was considered. The strength of the association is similar to that of a meta-analysis: 0.6% significant increase in AMI hospitalisations per 10 μg.m^−3^ increase in lag0 of PM_10_[5]. In contrast to the warm period, where no obvious association was observed, an IQR (10 μg.m^−3^) increase in lag0 of PM_10_ tended to increase AMI hospitalisations by 2% in the cold period, with borderline significance. The association between AMI hospitalisations and PM_10_ was strongest in the 1990–2000 period. This is supported by the association between the local fraction of PM_10_ (PM_rest_) and AMI hospitalisations in the cold period (2% increase). Reasons may include the possible differences in chemical composition and toxicity of PM_10_ in the warm and cold periods and during different time periods. In spring and summer dry road surfaces may increase the fraction of road dust in PM_10_, which may in turn be less toxic than combustion-related PM, which should be more common in winter-time. The mean PM_10_ level in our study (entire year average about 15 μg/m^3^) is much lower than PM_10_ in the most influential studies in the meta-analysis. It is, however, similar to recent population levels of PM_10_ in the Scandinavian countries, as shown in the ESCAPE study [39].

The strength of the association between AMI hospitalisations and PM_2.5_ (lag0) (1.7% per 10 μg.m^−3^ increase) is insignificant and somewhat weaker than that of a meta-analysis of 13 studies: 2.5% significant increase in AMI hospitalisations per 10 μg.m^−3^ increase in lag0 of PM_2.5_[5]. No significant associations were observed when stratified in the warm and cold periods.

Regarding rural background soot levels, a surrogate for LRT air pollution, no clear association was observed with AMI hospitalisation during any seasonal or year periods. A study from Germany (1999–2003) reported a significant increase in AMI hospitalisations with increasing levels of estimated personal soot exposure (relative risk 1.3 per 1.1 m^−1^ × 10^−5^) [40], but not with ambient soot. There might be an association between urban soot and AMI hospitalisations, but data on urban soot levels are not available for Gothenburg.

A recent WHO report discussed the current toxicological and epidemiological evidence regarding secondary aerosols [7]. The expert group proposed that inorganic secondary aerosols, such as ammonium, sulphates and nitrates, are valuable additional air quality metric to evaluate the health risks. We found, however, no associations for total nitrates or total ammonium ions. We observed an increase of 3% in AMI hospitalisations in the warm period during 1985–2000 per IQR increase in sulphate ions (lag0). One of the few epidemiological studies reported a borderline increase of 0.6% and 2.0% in CVD emergency room hospitalisations with an increase in sulphate (in PM_2.5_ fraction at urban sites, lag0) in the warm (April − September) and cold (October − March) periods, respectively [41]. If sulphate ions are a surrogate for the LRT PM, then the association between sulphate ions and AMI hospitalisations in the warm period (1985–2000) is in contrast with those observed for PM_10_ and PM_rest_ in the cold period (1990–2000). Hence, the local fraction of PM may be more important for AMI hospitalisations in the cold period, whilst the LTR fraction is of importance in the warm period.

The influence of geographical air mass origin on human health effects has been investigated sporadically, but never in a case-crossover design over a long study period [17]. We compared the likelihood of AMI hospitalisations for six different trajectories using Southern Scandinavia as the reference. The geographical air mass origin variable is not just a surrogate for LRT air pollution and its composition, but a combination of both the large-scale meteorological conditions as well as the large-scale air pollution transportation pattern. PM levels, including also sulphates and soot, were higher when air masses originated from Eastern Europe. The fact that NO_2_ and NO_x_ were higher when the air masses originated from Northern Europe could be due to lower wind speeds favouring the influence of locally emitted NO_x_.

We observed the largest increase in AMI hospitalisations at lag1 for air masses from Northern Scandinavia, UK/DK/North Sea and North Atlantic during the 2001–2010 in the cold period, although there was no significant difference between the seasons. This is in conflict with the association between PM_10_ and PM_rest_, and AMI hospitalisations, where the strongest associations were observed in the cold period in the time period (1990–2000) and not during 2001–2010, and we cannot provide any logical explanation for this.

In contrast no clear increase in AMI hospitalisations was observed when the air mass originated in Eastern Europe, where coal heating continues to dominate [11]. The adverse association at lag1 when the air mass originated in Northern Scandinavia (more days with effective local dispersion) might be caused by LRT of wood smoke particles in the cold season from rural areas. Both PM_10_ and PM_rest_ average levels were much higher in 2001–2010 than in 1990–2000 for air masses originated from Northern Scandinavia which can be caused by the increased use of wood burning for heating in Sweden [42].

The classification according to local meteorology (e.g. temperature inversions) indicated that days with limited local dispersion had a strong association with AMI hospitalisations at lag0 during 2001–2010 in the cold period. This is in accordance with the positive association in the same periods between air masses (UK/DK/North Sea and North Atlantic) having rather high percentage of days with limited local dispersion and thus more influence from local emissions. This indicates significant contribution of local emissions within Gothenburg.

Advantages of our study include accurate meteorological, air pollution and AMI hospitalisation data. Data from the Swedish Hospital Registry have high validity for a diagnosis of MI [43]. Autopsy rates for persons dying outside hospital from IHD are high for younger, but not older people [44]. Some disease misclassification is possible, but it is unlikely to be related to air pollution levels and the geographical origin of air masses. Our study period of 26 years is longer than the other studies that investigated the association between air pollution levels and AMI, which had study periods of 2–16 years [5]. Unlike our study, most studies do not report the actual number of cases or days included in their case-crossover analyses, due to missing exposure data.

Another advantage of our study is that unlike most of the other studies, we investigated the effect of seasonal and time periods by interaction analyses. We also investigated a more refined PM estimate (PM_rest_), geographical air mass origins and limited/effective local dispersion days. To our knowledge no similar classifications have been used before in health-related studies.

As with all ecological epidemiology study designs, a disadvantage of our study is a possible exposure misclassification, i.e. the assumption that the ambient temperature, humidity and air pollution measured in the city are the same across Gothenburg. The exposure error resulting from using ambient temperature and air pollution as a surrogate for personal exposure can potentially lead to bias in the estimated association, and this can be more pronounced among the elderly and other frail groups who generally spend most of their time indoors. However, in a case-crossover study investigating the risk of AMI, more refined exposure PM_2.5_ surrogates that account for human activities did not result in larger health effects compared with analyses that used a central monitoring site [45].

A second limitation is the inability to consider the chemical composition of PM_10_ and PM_2.5_ and the relatively short measurement period of PM_2.5_ (only 5 years) [34].

A third limitation is that of the uncertainties of trajectory models, which have been discussed in many studies [46,47]. The sources of uncertainty for a single trajectory can be the meteorological field, starting height, trajectory calculation or trajectory clustering. In this study, an ensemble method was carried out to diminish the uncertainty (as regards starting height, trajectory calculation) of a single trajectory [19]. However, the uncertainties from other sources cannot be avoided.

A fourth limitation is that information on effect modifiers, e.g. the use of medications, pre-existing CVD or comorbidities, was not available in our study [48-50]. Such effect modifiers may bias the association between the air pollutants and AMI hospital admissions in either direction.

We performed a large number of tests which increases the probability of obtaining spurious significant associations. When multiple hypotheses are tested, the risk of false positive conclusions can increase and be higher than the intended level of e.g. 0.05. There are various ways of controlling the family-wise error rate (the risk of at least one false positive), by e.g. Bonferroni correction, the Holm step-down procedure or splitting and recycling the test mass [51,52]. However, our study is very exploratory and can be considered as hypothesis-generating where one aim is to find patterns for how the composition of air masses from different geographical origins is associated with AMI. The p-values can be considered descriptive rather than decisive and the results can be used as a guide for a future study that can focus on the cold season − where this study showed the strongest results.

AMI hospitalisations may not accurately reflect AMI incidence. Fatal coronary heart disease events that occurred out-of-hospital could have been included in our analyses. Mortality in AMI has decreased much more (by about 50% in the past 15 years) than incidence of AMI (about 16%) [53]. Much of the decrease in mortality is, however, caused by better in-hospital treatment, and nearly one in three patients with a major coronary heart disease event dies without reaching a hospital. Nevertheless, AMI hospitalisations are a reasonable surrogate for major coronary heart disease events.

## Conclusions

Locally generated PM (PM_rest_) is associated with an increase in AMI hospitalisations during 1990–2000 in the cold period, whilst days with limited local dispersion within Gothenburg were important during 2001–2010, also in the cold period. Altogether this indicates the significance of local emissions from e.g. traffic within Gothenburg.

## Abbreviations

AMI: Acute myocardial infarction; CA: Cumulative average; CVD: Cardiovascular disease; EU: European union; ICD: International classification of diseases; IHD: Ischemic heart disease; IQR: Inter-quartile range; LRT: Long-range transported; NO_x_: Nitrogen oxides; NO_2_: Nitrogen dioxide; O_3_: Ground-level ozone; OR: Odds ratios; PM_ion_: Sum of sulphate, nitrate and ammonium; PM_rest_: Difference between urban PM_10_ and rural PM_ion_ was a surrogate for locally generated PM_10_; PM_2.5_: Particulate matter with an aerodynamic diameter smaller than 2.5 μm; PM_10_: Particulate matter with an aerodynamic diameter smaller than 10 μm; 95% CI: 95% confidence interval; WHO: World Health Organisation.

## Competing interests

The authors declare that they have no competing interests. The Swedish Research Council Formas funded the study. The funders had no role in study design, data collection and analysis, decision to publish, or preparation of the manuscript.

## Authors’ contributions

JW, EA, LB and GS designed the study. AR cleaned and contributed the acute myocardial infarction hospital admission data. KS, MHE and LT provided and ensured the quality of the air pollution and meteorological data. JW analysed the data. All authors contributed to writing and revising the manuscript and approved the final manuscript.

## Supplementary Material

Additional file 1 Examples of typical pathways of air masses in Gothenburg, Sweden. Click here for file

Additional file 2 Time-series of acute myocardial infarction hospitalisation in Gothenburg, Sweden (1 January 1985 − 31 December 2010). Click here for file

Additional file 3 **Time-series of available PM**_**10**_**urban levels in Gothenburg, Sweden during the study period 1 January 1990 − 31 December 2010.** Click here for file

Additional file 4 **(a) Average PM**_10_*, PM_**ion**_**, PM**_rest_*, PM_2.5_*, soot, temperature and wind speed and (b) NO_**x**_**, NO**_**2**_**, O**_**3**_**and relative humidity levels by origin of air mass in Gothenburg, Sweden (1 January 1985 − 31 December 2010).** Click here for file

Additional file 5 **(a) Average PM**_**ion**_**, sulphate, total nitrate and total ammonium by origin of air mass in Gothenburg, Sweden (1 January 1985 − 31 December 2010).** Click here for file

Additional file 6 Association between the lag0, lag1 and the 2-day cumulative average of sulphate, total nitrate, total ammonium and acute myocardial infarction hospitalisation in Gothenburg (1985−2010) as percentage change in risk (%) and 95% confidence intervals during (a) the entire year, (b) warm period (April − September) and (c) cold period (October − March). Click here for file

Additional file 7 Unadjusted association between the lag0 and lag1 of the origin of the air masses and acute myocardial infarction hospitalisation in Gothenburg during the entire year (1985−2010) as percentage change in risk (%) and 95% confidence intervals. Click here for file

Additional file 8 Association between the lag0 and lag1 of the origin of the air masses and acute myocardial infarction hospitalisations in Gothenburg, Sweden as percentage change in risk (%) and 95% confidence intervals during the cold period (October−March) for (a) 1985−2010, (b) 1985−2000 and (c) 2001−2010. Click here for file

Additional file 9 **Average PM**_**10 **_**and PM**_**rest**_**levels by origin of air mass in Gothenburg, Sweden by (a,d) the entire year, (b,e) warm (April − September) and (c,f) cold periods (October − March). **These periods were further stratified by 1990−2010, 1990−2000 and 2001−2010. Click here for file

Additional file 10 **Descriptive statistics for daily PM**_10_*, PM_**ion**_**, PM**_rest_*, PM_2.5_* and soot levels (lag0) in Gothenburg, Sweden by seasonal and year periods (1 January 1985−31 December 2010). Click here for file
