# Supplementary figures and images for: The effect of secondary inorganic aerosols, soot and the geographical origin of air mass on acute myocardial infarction hospitalisations in Gothenburg, Sweden during 1985–2010: a case-crossover study
Source: Environ Health. 2014 Jul 29;13:61. doi: 10.1186/1476-069X-13-61 (PMC4131776; doi:10.1186/1476-069X-13-61)

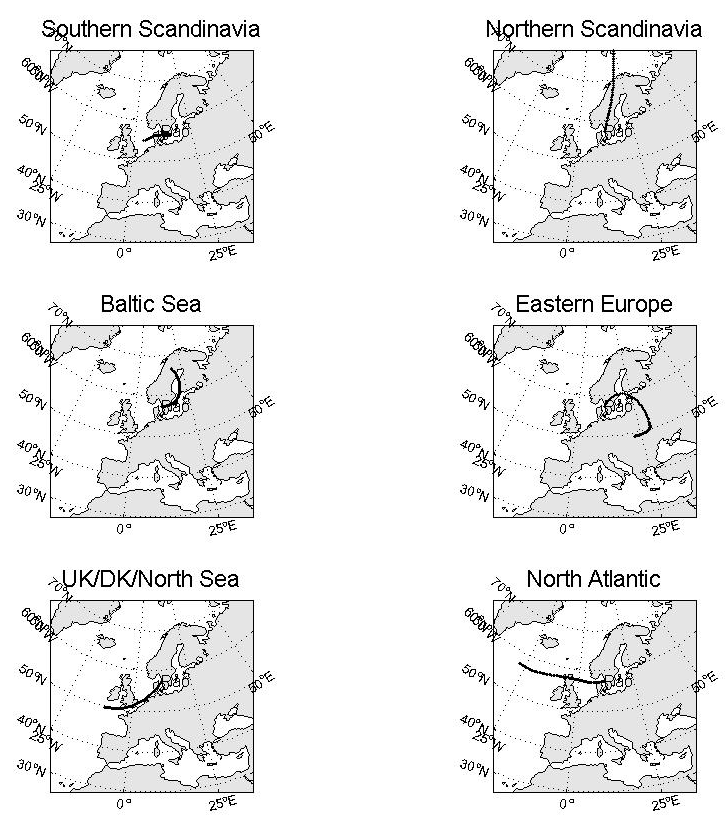


**Additional file 1. Examples of typical pathways of air masses in Gothenburg, Sweden.**

Supplement: Additional file 1 — Examples of typical pathways of air masses in Gothenburg, Sweden. [file 1476-069X-13-61-S1.docx]
