# Supplementary material for: The effect of secondary inorganic aerosols, soot and the geographical origin of air mass on acute myocardial infarction hospitalisations in Gothenburg, Sweden during 1985–2010: a case-crossover study
Source: Environ Health. 2014 Jul 29;13:61. doi: 10.1186/1476-069X-13-61 (PMC4131776; doi:10.1186/1476-069X-13-61)
Supplement: Additional file 2 — Time-series of acute myocardial infarction hospitalisation in Gothenburg, Sweden (1 January 1985 − 31 December 2010). [file 1476-069X-13-61-S2.docx]

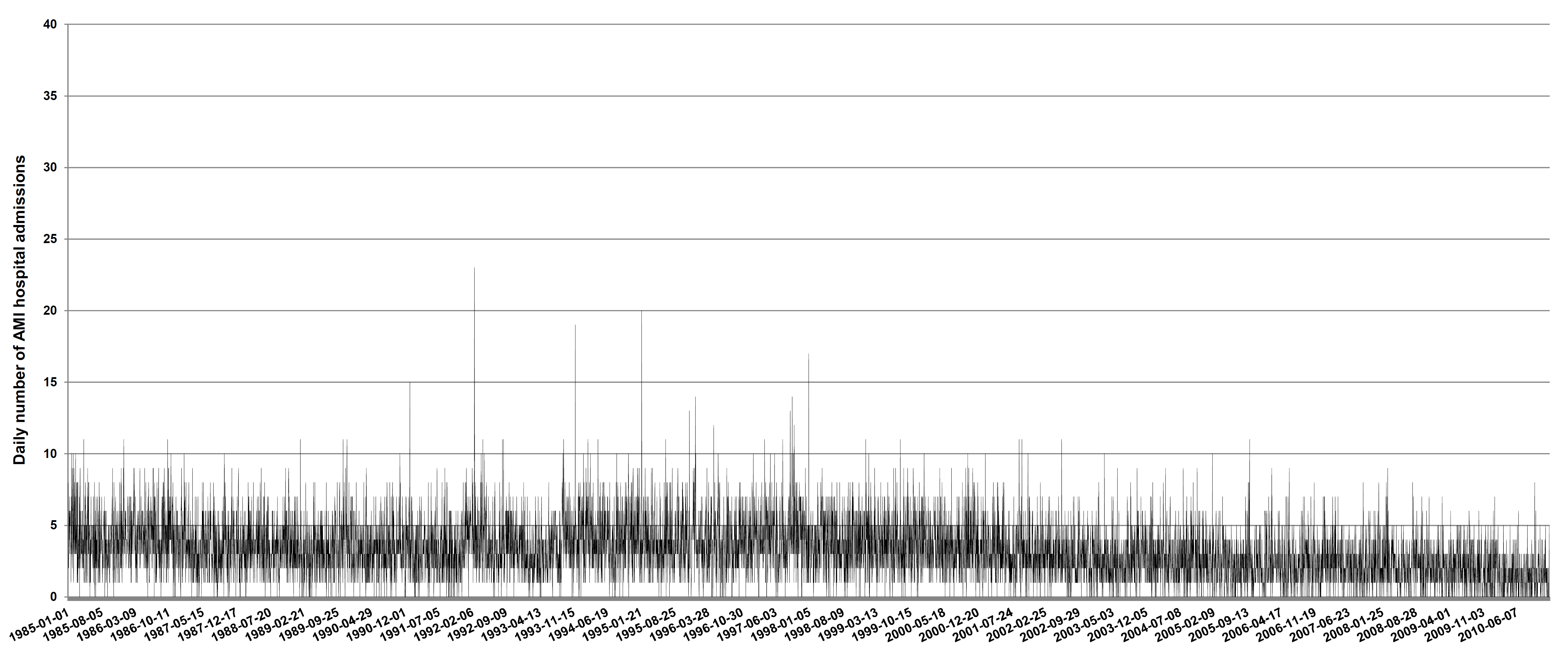


**Additional file 2. Time-series of acute myocardial infarction hospitalizations in Gothenburg, Sweden (1 January 1985 − 31 December 2010).**
