# Supplementary material for: The effect of secondary inorganic aerosols, soot and the geographical origin of air mass on acute myocardial infarction hospitalisations in Gothenburg, Sweden during 1985–2010: a case-crossover study
Source: Environ Health. 2014 Jul 29;13:61. doi: 10.1186/1476-069X-13-61 (PMC4131776; doi:10.1186/1476-069X-13-61)
Supplement: Additional file 3 — Time-series of available PM 10 urban levels in Gothenburg, Sweden during the study period 1 January 1990 − 31 December 2010. [file 1476-069X-13-61-S3.docx]

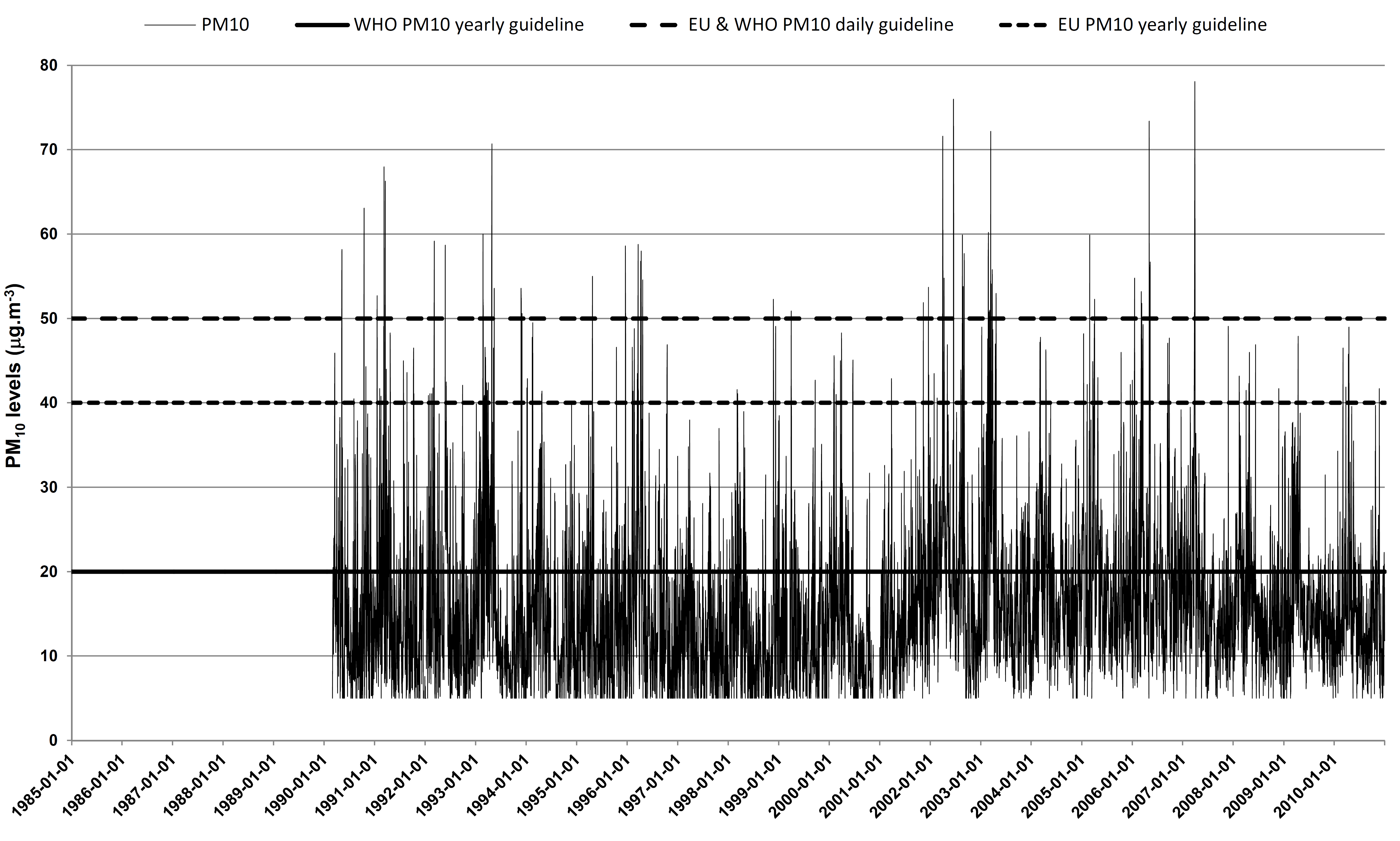


**Additional file 3. Time-series of available PM_10_ urban levels in Gothenburg, Sweden during the**

**study period 1 January 1990 − 31 December 2010.**
