# Supplementary material for: The effect of secondary inorganic aerosols, soot and the geographical origin of air mass on acute myocardial infarction hospitalisations in Gothenburg, Sweden during 1985–2010: a case-crossover study
Source: Environ Health. 2014 Jul 29;13:61. doi: 10.1186/1476-069X-13-61 (PMC4131776; doi:10.1186/1476-069X-13-61)
Supplement: Additional file 4 — (a) Average PM 10 *, PM ion , PM rest *, PM 2.5 *, soot, temperature and wind speed and (b) NO x , NO 2 , O 3 and relative humidity levels by origin of air mass in Gothenburg, Sweden (1 January 1985 − 31 December 2010). [file 1476-069X-13-61-S4.docx]

A


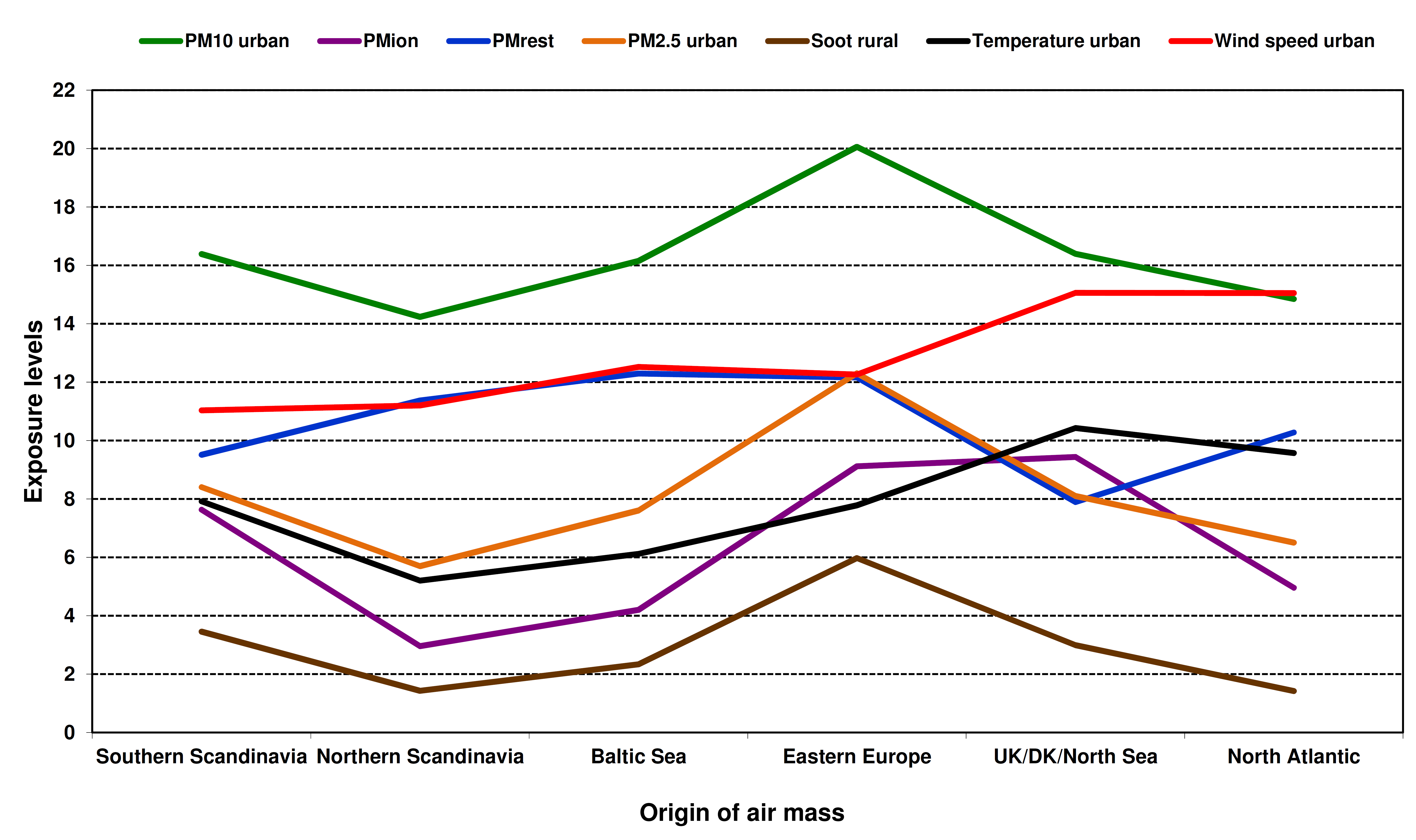


B


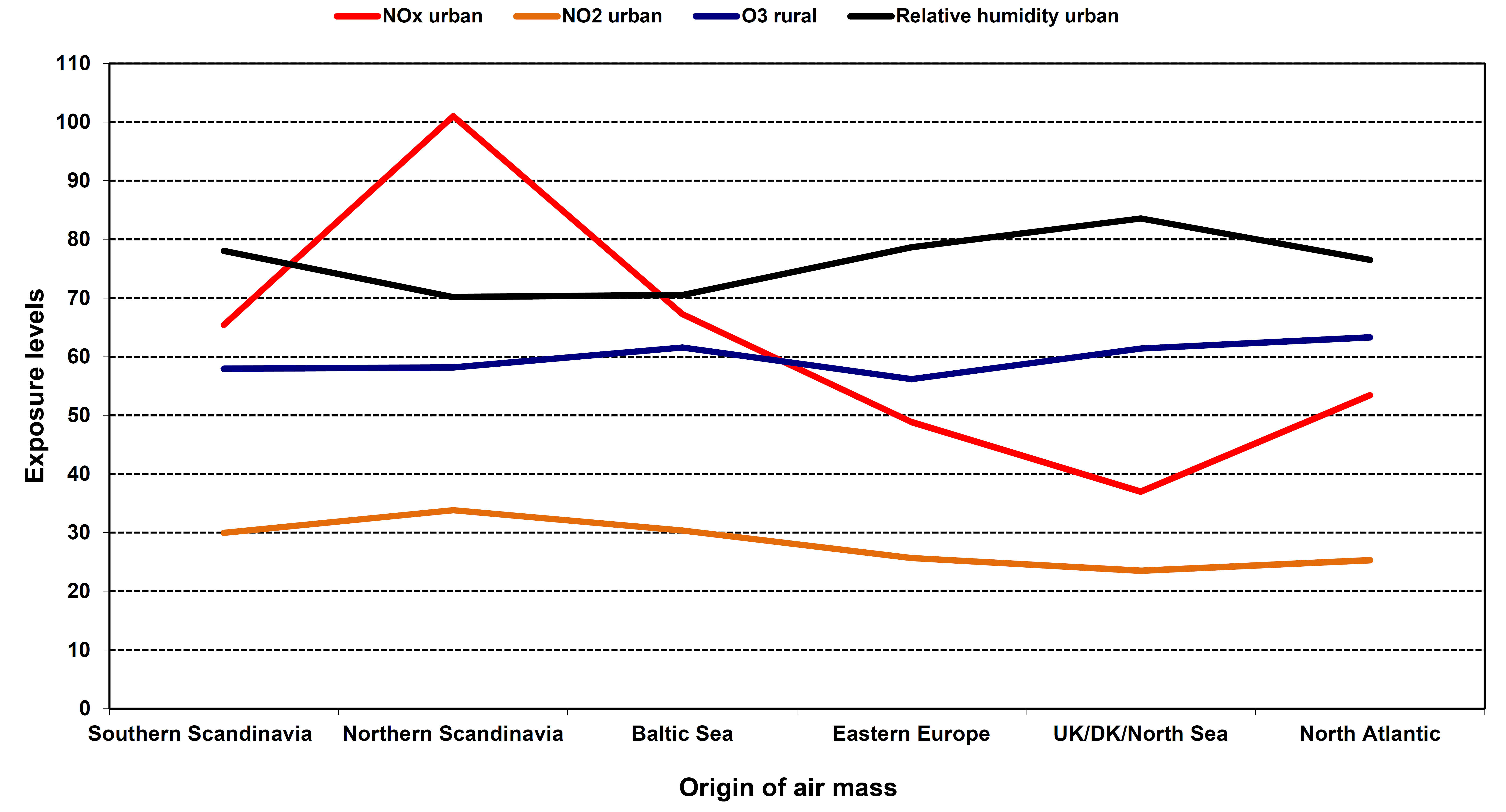


**Additional file 4. (a) Average PM_10_*, PM_ion_, PM_rest_*, PM_2.5_*, soot, temperature and wind speed and (b) NO_x_, NO_2_, O_3_** **and relative humidity** **levels by origin of air mass in Gothenburg, Sweden (1 January 1985 − 31 December 2010).**

* PM_10_ and PM_rest_ data available from 1990-2010 and PM_2.5_ data available from 2006-2010

Pollutant levels in μg.m^-3^, temperature in °C, relative humidity in % and wind speed in km.h^-1^
