# Supplementary material for: The effect of secondary inorganic aerosols, soot and the geographical origin of air mass on acute myocardial infarction hospitalisations in Gothenburg, Sweden during 1985–2010: a case-crossover study
Source: Environ Health. 2014 Jul 29;13:61. doi: 10.1186/1476-069X-13-61 (PMC4131776; doi:10.1186/1476-069X-13-61)
Supplement: Additional file 6 — Association between the lag0, lag1 and the 2-day cumulative average of sulphate, total nitrate, total ammonium and acute myocardial infarction hospitalisation in Gothenburg (1985−2010) as percentage change in risk (%) and 95% confidence intervals during (a) the entire year, (b) warm period (April − September) and (c) cold period (October − March). [file 1476-069X-13-61-S6.docx]

A


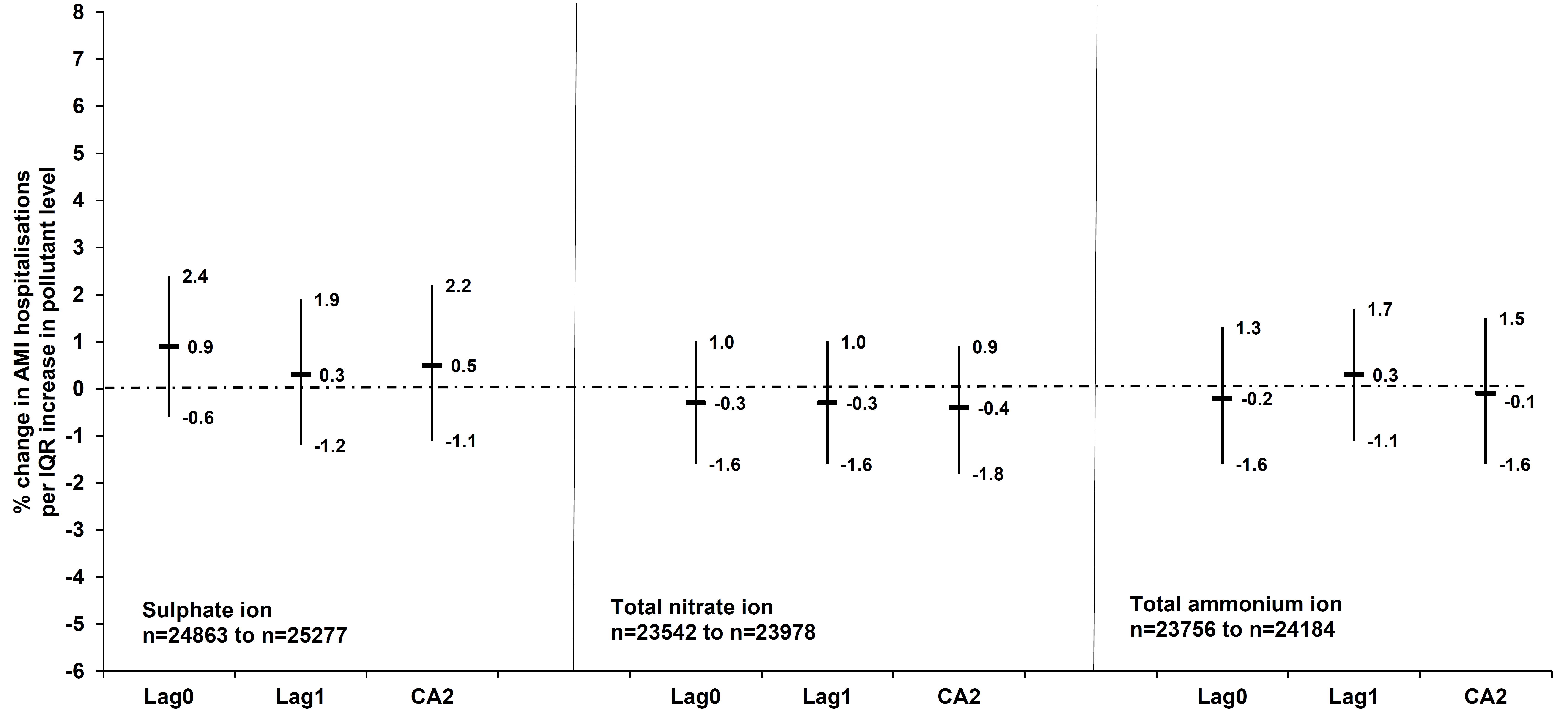


B


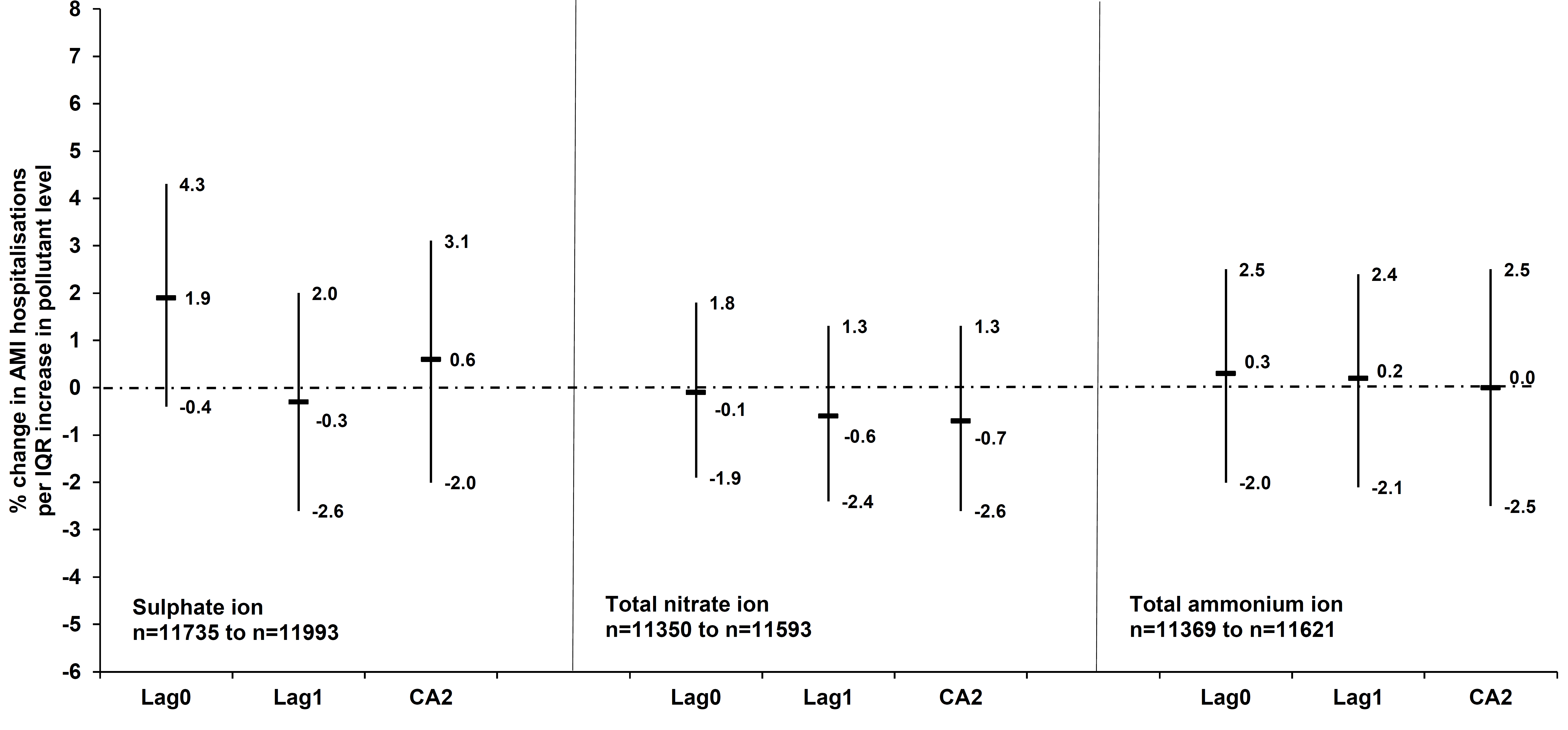


C


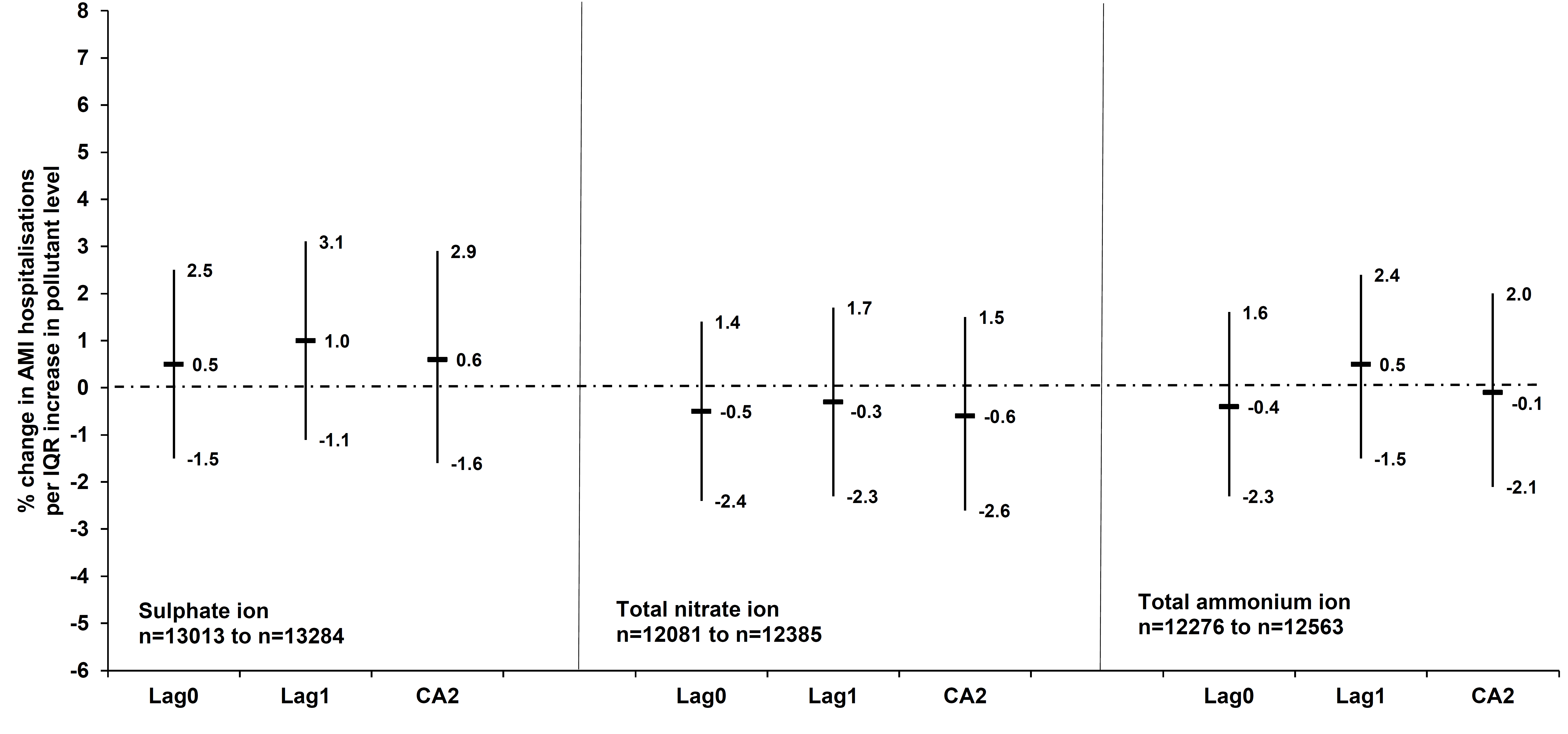


**Additional file 6. Association between the lag0, lag1 and the 2-day cumulative average of sulphate, total nitrate, total ammonium and acute myocardial infarction hospitalizations in Gothenburg (1985−2010) as percentage change in risk (%) and 95% confidence intervals during (a) the entire year, (b) warm period (April−September)and (c) cold period (October−March).**

Models adjusted for temperature, relative humidity and public holidays. Number of cases (n) used in the models is less than the original number due to missing exposure data
