# Supplementary material for: The effect of secondary inorganic aerosols, soot and the geographical origin of air mass on acute myocardial infarction hospitalisations in Gothenburg, Sweden during 1985–2010: a case-crossover study
Source: Environ Health. 2014 Jul 29;13:61. doi: 10.1186/1476-069X-13-61 (PMC4131776; doi:10.1186/1476-069X-13-61)
Supplement: Additional file 7 — Unadjusted association between the lag0 and lag1 of the origin of the air masses and acute myocardial infarction hospitalisation in Gothenburg during the entire year (1985−2010) as percentage change in risk (%) and 95% confidence intervals. [file 1476-069X-13-61-S7.docx]

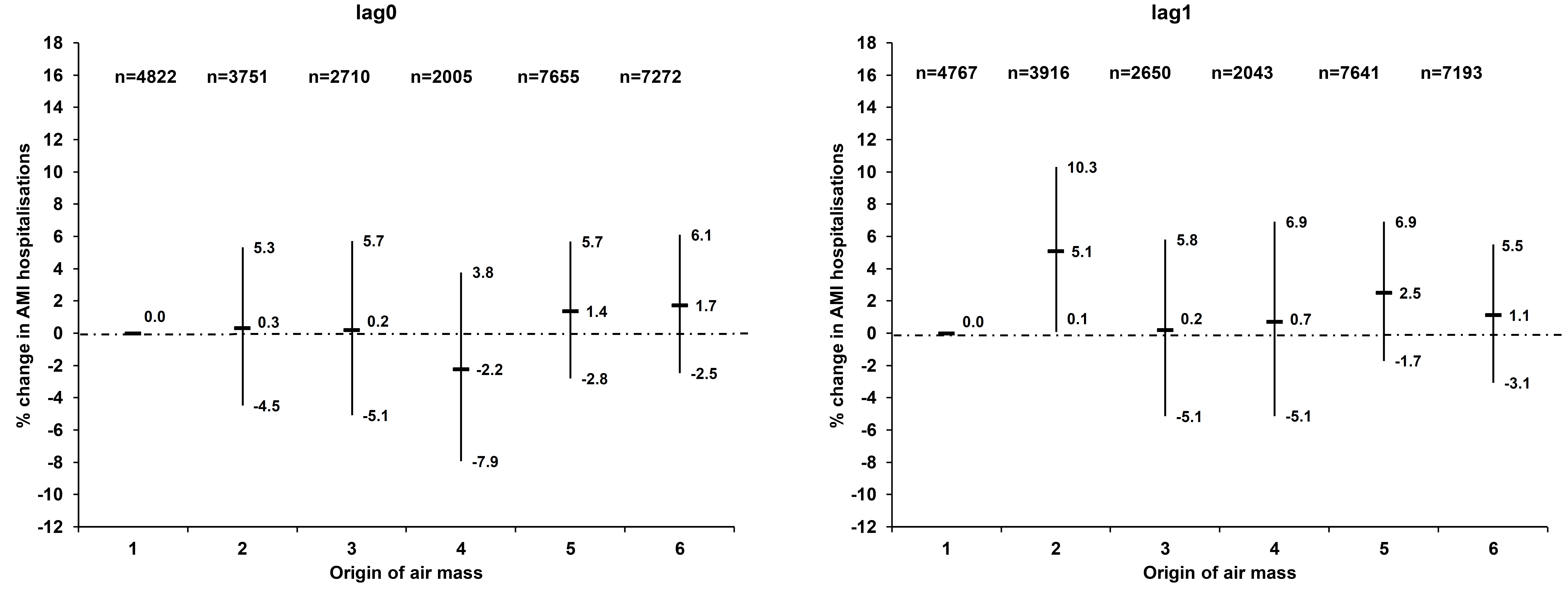


**Additional file 7. Unadjusted association between the lag0 and lag1 of the origin of the air masses and acute myocardial infarction hospitalizations in Gothenburg during the entire year (1985−2010) as percentage change in risk (%) and 95% confidence intervals.**

Number of cases (n) used in the models is less than the original number due to missing exposure data

Origin of air masses: 1: Southern Scandinavia, 2. Northern Scandinavia, 3: Baltic Sea, 4: Eastern Europe, 5. UK/DK/North Sea and 6: North Atlantic

The reference category is 1: Southern Scandinavia
