# Supplementary material for: The effect of secondary inorganic aerosols, soot and the geographical origin of air mass on acute myocardial infarction hospitalisations in Gothenburg, Sweden during 1985–2010: a case-crossover study
Source: Environ Health. 2014 Jul 29;13:61. doi: 10.1186/1476-069X-13-61 (PMC4131776; doi:10.1186/1476-069X-13-61)
Supplement: Additional file 8 — Association between the lag0 and lag1 of the origin of the air masses and acute myocardial infarction hospitalisations in Gothenburg, Sweden as percentage change in risk (%) and 95% confidence intervals during the cold period (October−March) for (a) 1985−2010, (b) 1985−2000 and (c) 2001−2010. [file 1476-069X-13-61-S8.docx]

A


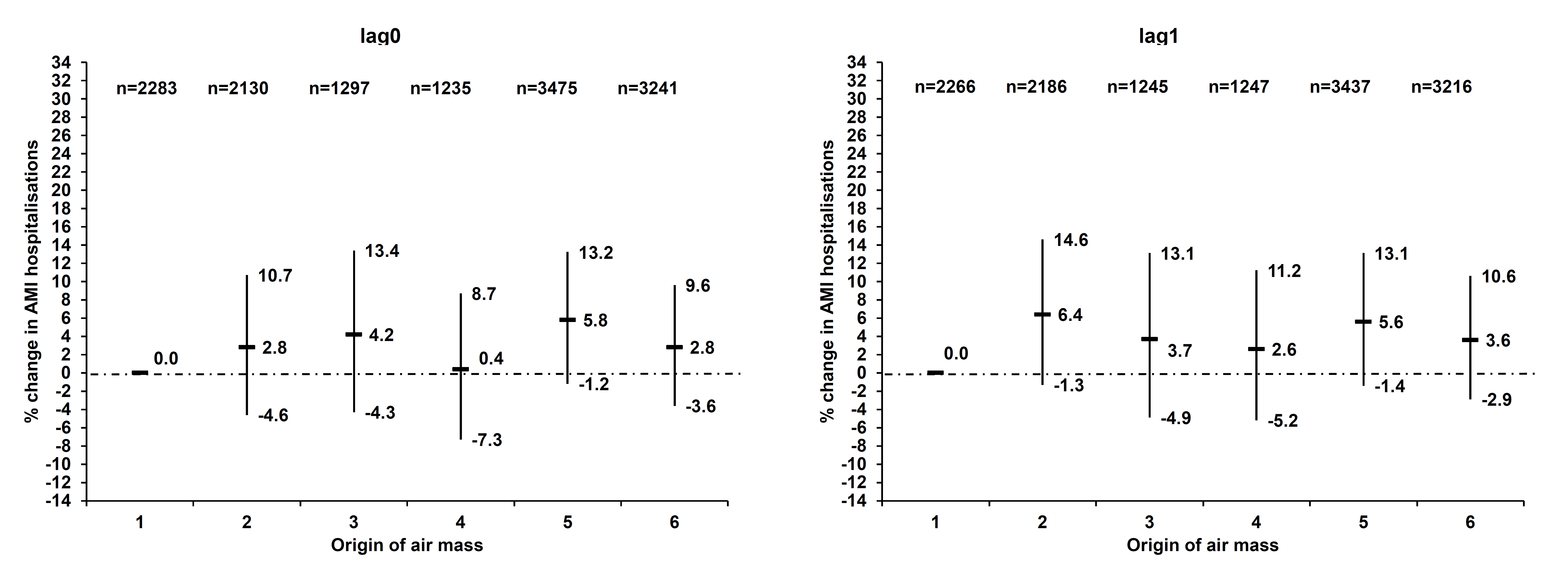


B


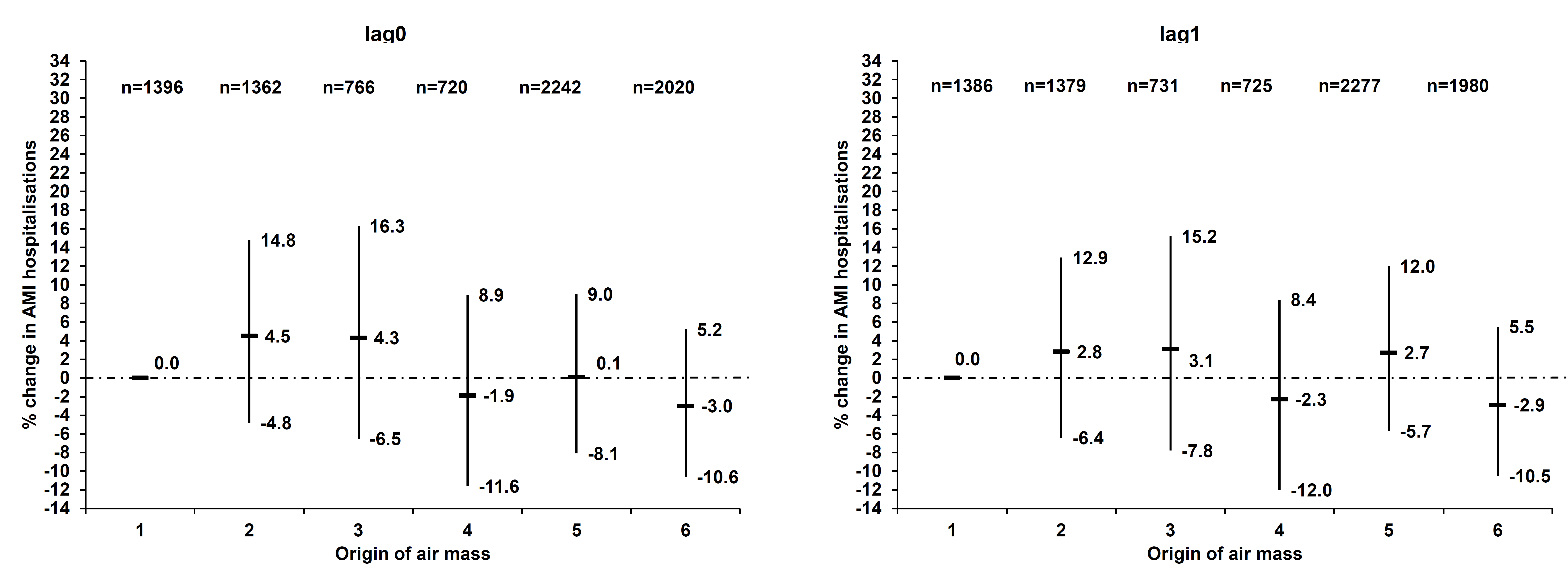


C


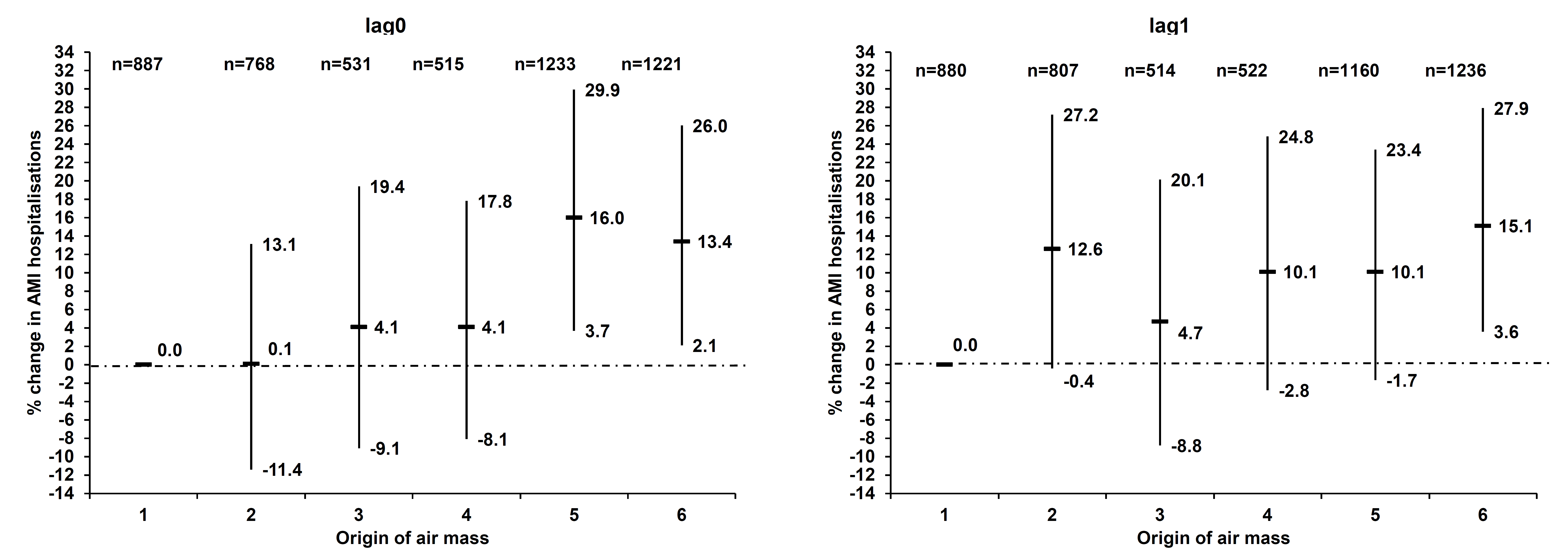


**Additional file 8.** Association between the lag0 and lag1 of the origin of the air masses and acute myocardial infarction hospitalisations in Gothenburg, Sweden as percentage change in risk (%) and 95% confidence intervals during the cold period (October−March) for (a) 1985−2010, (b) 1985−2000 and (c) 2001−2010.

Models adjusted for temperature, relative humidity and public holidays

Number of cases (n) used in the models is less than the original number due to missing exposure data

Origin of air masses: 1: Southern Scandinavia, 2. Northern Scandinavia, 3: Baltic Sea, 4: Eastern Europe, 5. UK/DK/North Sea and 6: North Atlantic. The reference category is 1: Southern Scandinavia
