# Supplementary material for: The effect of secondary inorganic aerosols, soot and the geographical origin of air mass on acute myocardial infarction hospitalisations in Gothenburg, Sweden during 1985–2010: a case-crossover study
Source: Environ Health. 2014 Jul 29;13:61. doi: 10.1186/1476-069X-13-61 (PMC4131776; doi:10.1186/1476-069X-13-61)
Supplement: Additional file 9 — Average PM 10 and PM rest levels by origin of air mass in Gothenburg, Sweden by (a,d) the entire year, (b,e) warm (April − September) and (c,f) cold periods (October − March). These periods were further stratified by 1990−2010, 1990−2000 and 2001−2010. [file 1476-069X-13-61-S9.docx]

| A 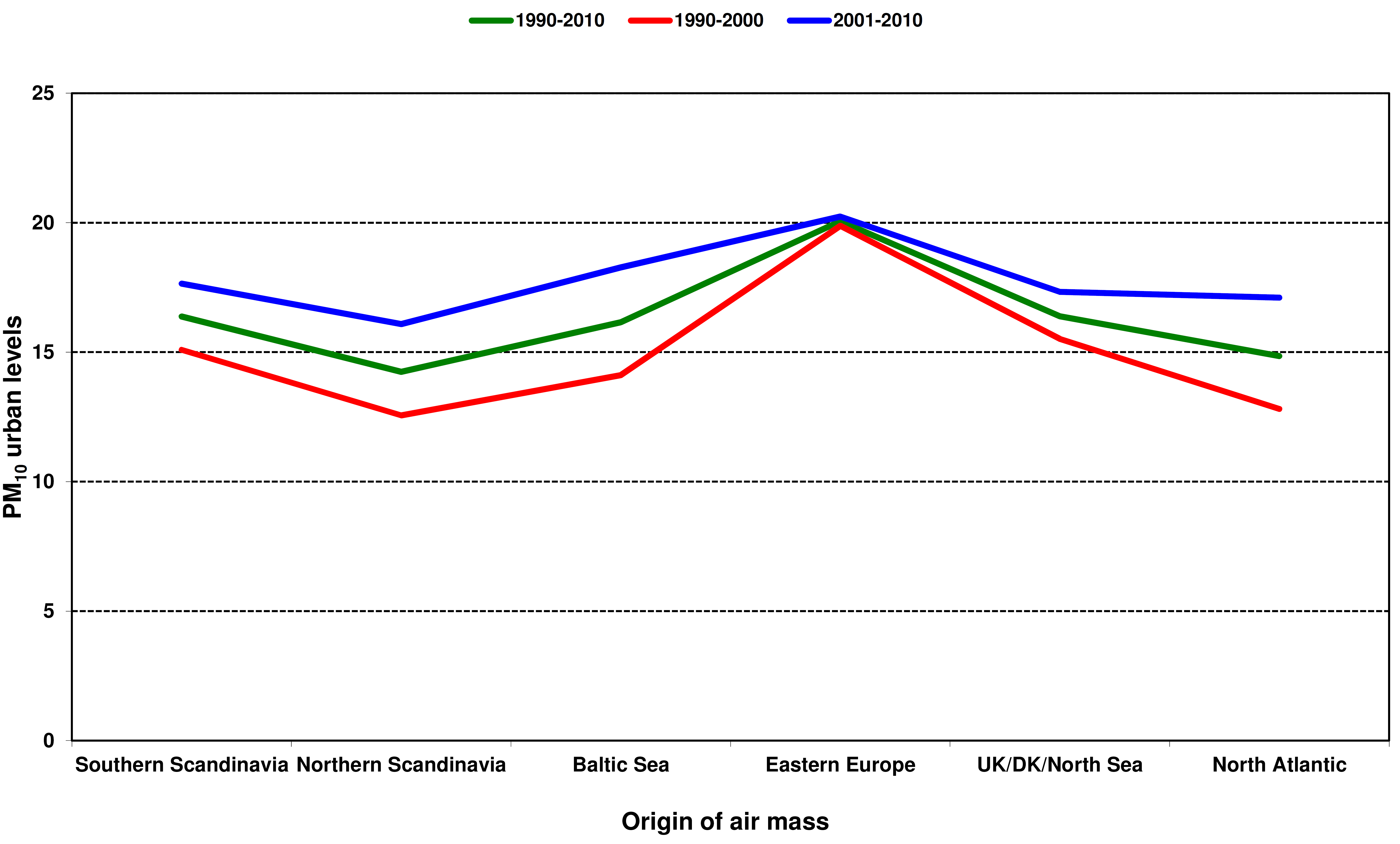 | B 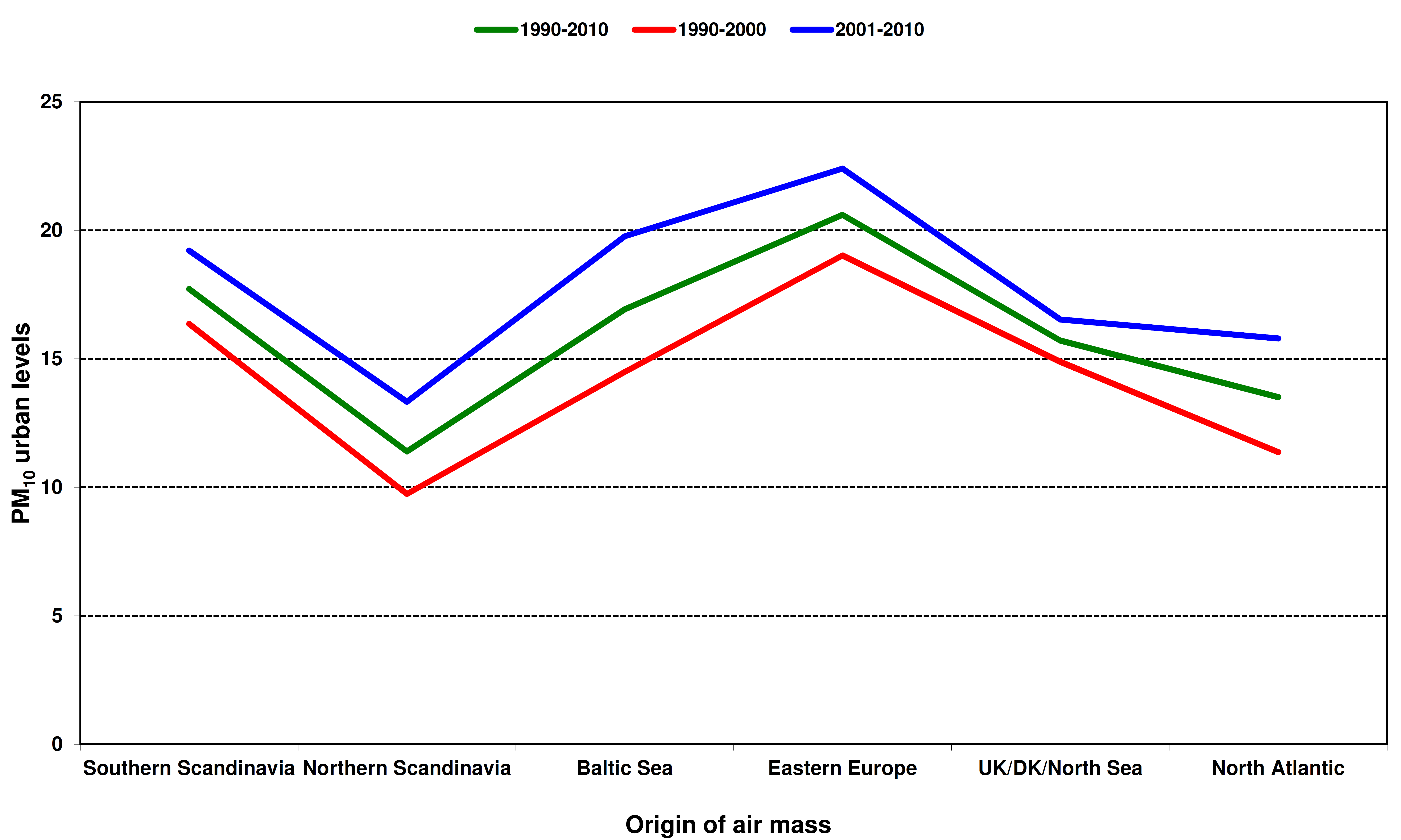 | C 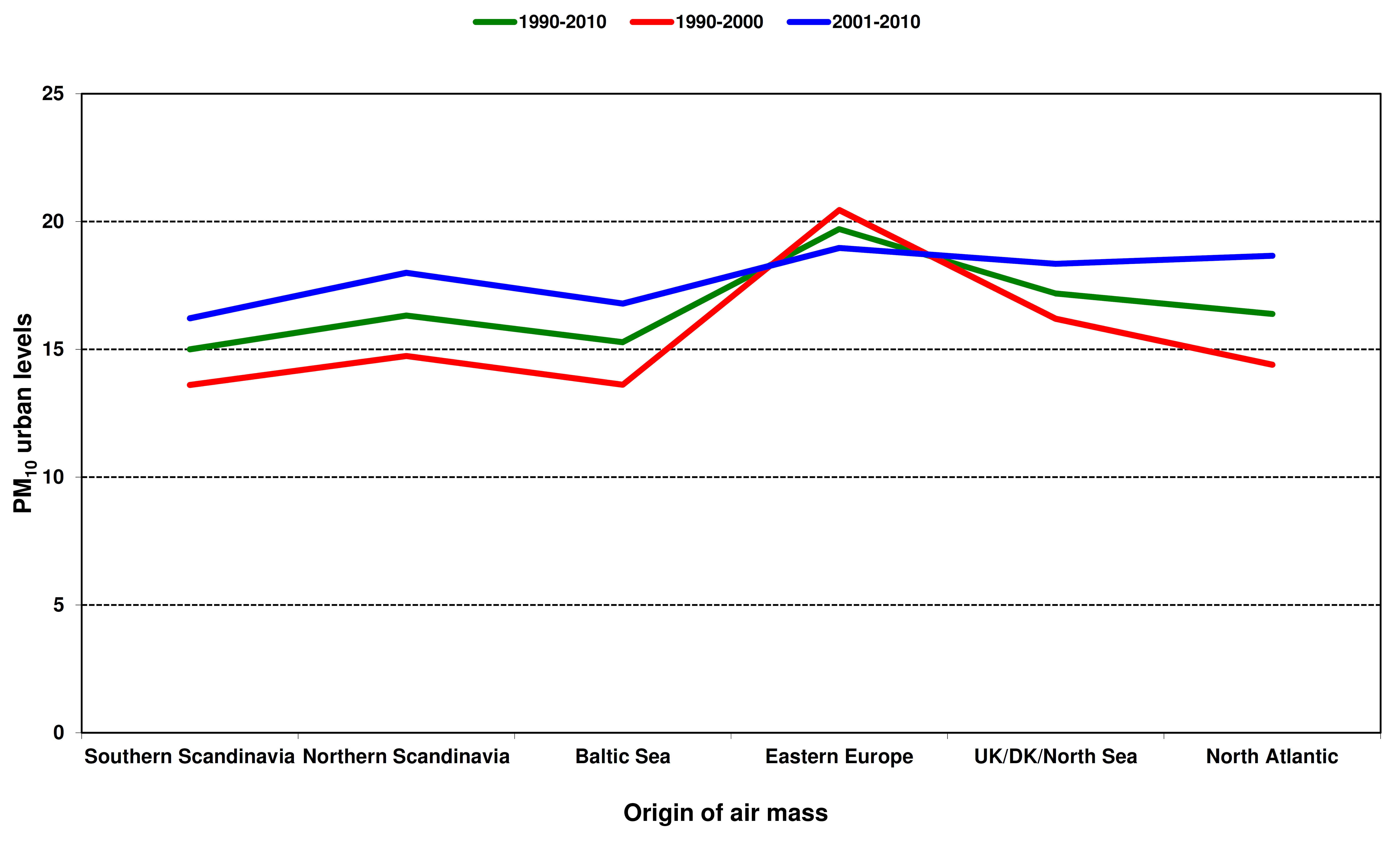 |
| --- | --- | --- |
| D 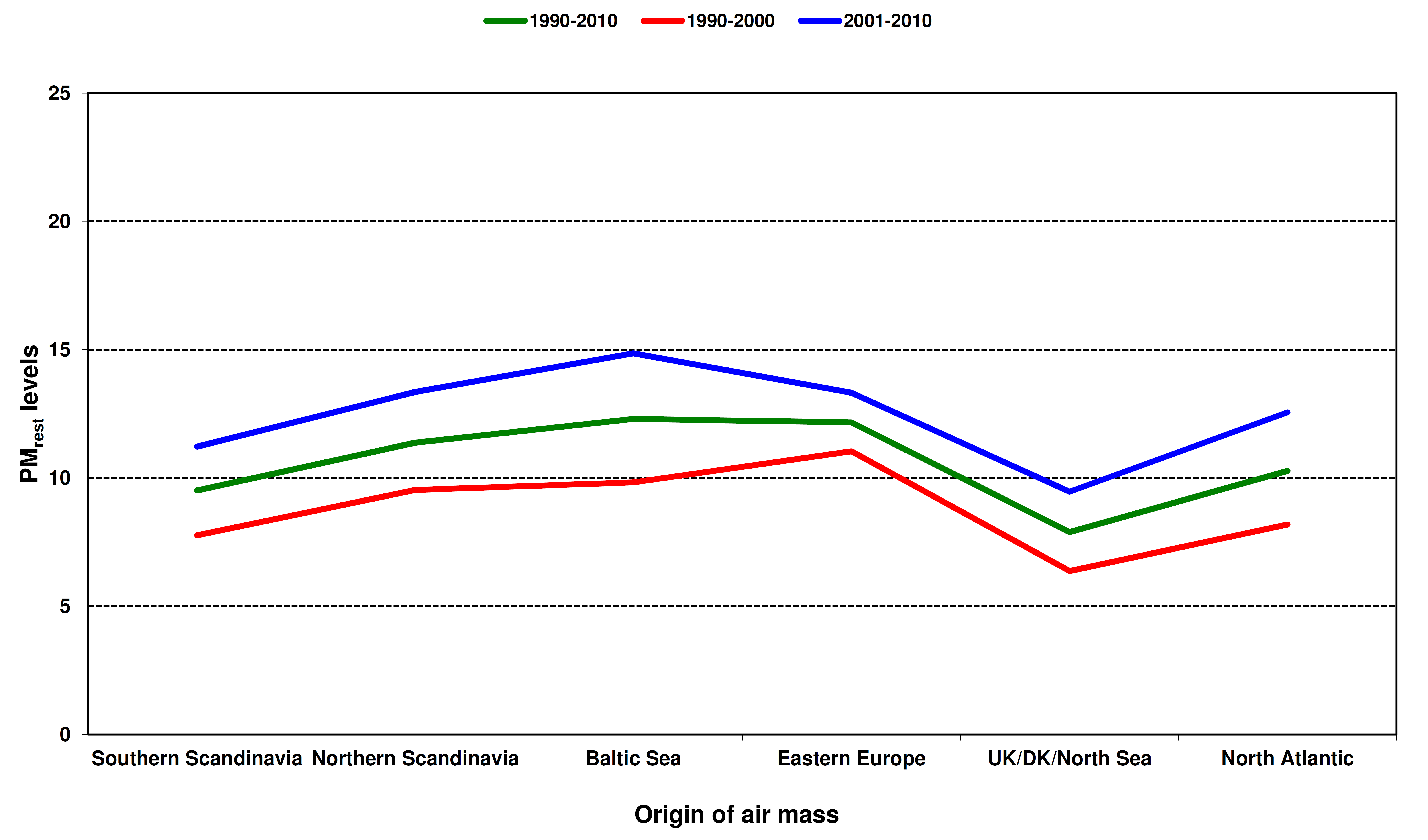 | E 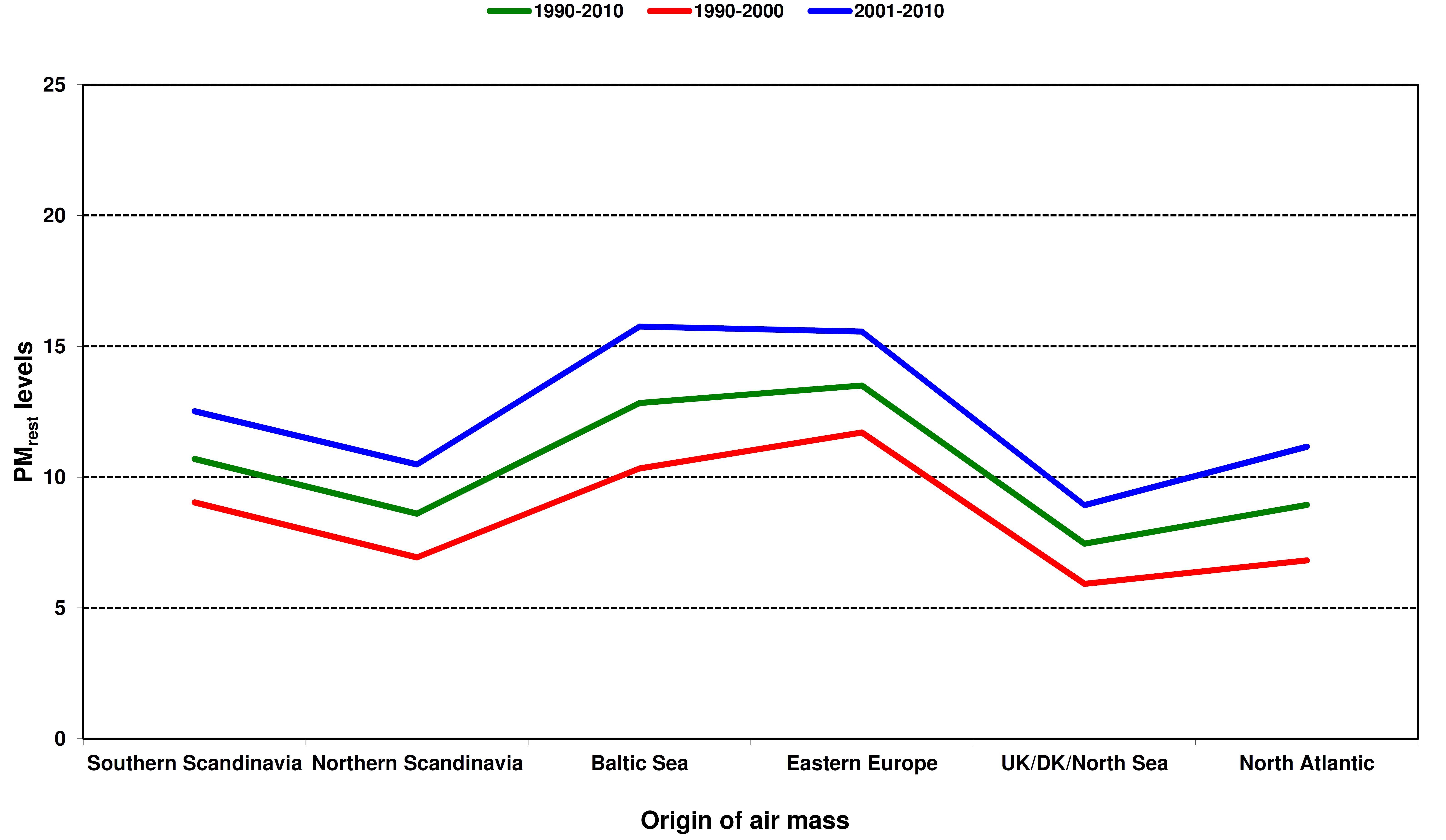 | F 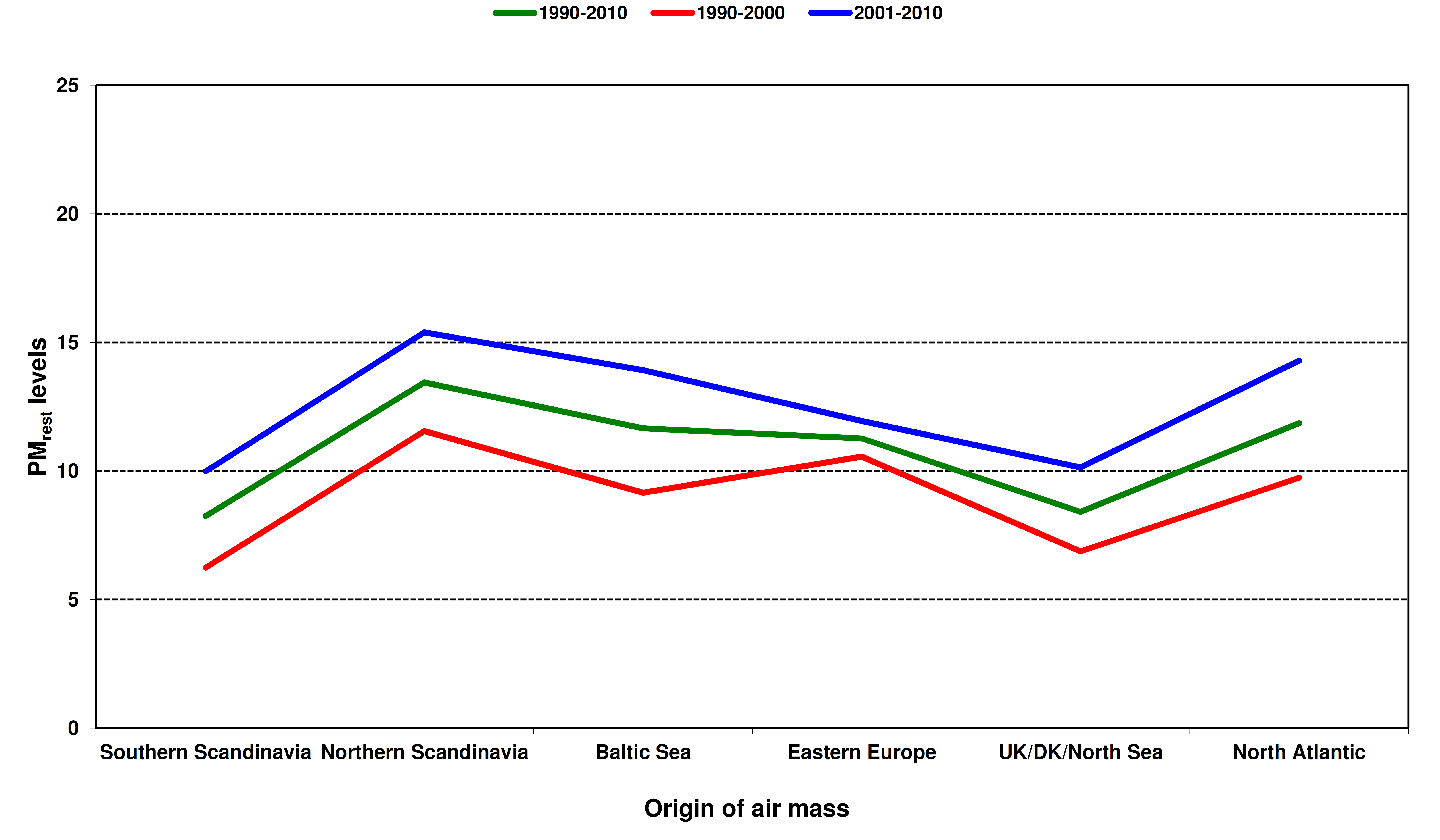 |

**Additional file 9. Average PM_10_ and PM_rest_** **levels by origin of air mass in Gothenburg, Sweden by (a,d) the entire year, (b,e) warm (April−September) and (c,f) cold periods (October−March). These periods were further stratified by 1990−2010, 1990−2000 and 2001−2010.**

* PM_10_ and PM_rest_ data available from 1990-2010

Pollutant levels in μg.m^-3^
