# Supplementary material for: The effect of secondary inorganic aerosols, soot and the geographical origin of air mass on acute myocardial infarction hospitalisations in Gothenburg, Sweden during 1985–2010: a case-crossover study
Source: Environ Health. 2014 Jul 29;13:61. doi: 10.1186/1476-069X-13-61 (PMC4131776; doi:10.1186/1476-069X-13-61)
Supplement: Additional file 10 — Descriptive statistics for daily PM 10 *, PM ion , PM rest *, PM 2.5 * and soot levels (lag0) in Gothenburg, Sweden by seasonal and year periods (1 January 1985−31 December 2010). [file 1476-069X-13-61-S10.docx]

**Additional file 10. Descriptive statistics for daily PM_10_*, PM_ion_, PM_rest_*, PM_2.5_* and soot levels (lag0) in Gothenburg, Sweden by seasonal and year periods (1 January 1985−31 December 2010).**

|  | **No. days missing data** | **Mean** | **SD** | **Min** | **Max** |
| --- | --- | --- | --- | --- | --- |
| **Warm period, 1985-2000 (2928 days)** |  |  |  |  |  |
| PM_10_ (µg.m^-3^) | 966 | 13.8 | 8.6 | 5.0 | 70.7 |
| PM_ion_ (µg.m^-3^) | 386 | 7.0 | 5.7 | 0.5 | 63.6 |
| PM_rest_ (µg.m^-3^) | 1052 | 7.7 | 6.6 | 0.0 | 53.6 |
| PM_2.5_ (µg.m^-3^) | 2928 | - | - | - | - |
| Soot (µg.m^-3^) | 140 | 2.0 | 2.4 | 0.8 | 23.7 |
| **Warm period, 2001-2010 (1830 days)** |  |  |  |  |  |
| PM_10_ (µg.m^-3^) | 7 | 17.1 | 8.6 | 5.0 | 76.0 |
| PM_ion_ (µg.m^-3^) | 47 | 5.7 | 4.0 | 0.5 | 61.9 |
| PM_rest_ (µg.m^-3^) | 54 | 11.4 | 7.6 | 0.1 | 65.0 |
| PM_2.5_ (µg.m^-3^) | 917 | 7.2 | 4.2 | 0.9 | 35.6 |
| Soot (µg.m^-3^) | 28 | 1.2 | 1.2 | 0.8 | 14.9 |
| **Cold period, 1985-2000 (2916 days)** |  |  |  |  |  |
| PM_10_ (µg.m^-3^) | 1022 | 15.3 | 9.8 | 5.0 | 68.0 |
| PM_ion_ (µg.m^-3^) | 365 | 7.5 | 6.6 | 0.4 | 63.6 |
| PM_rest_ (µg.m^-3^) | 1,157 | 8.8 | 8.1 | 0.0 | 56.9 |
| PM_2.5_ (µg.m^-3^) | 2916 | - | - | - | - |
| Soot (µg.m^-3^) | 120 | 4.3 | 5.3 | 0.8 | 48.2 |
| **Cold period, 2001-2010 (1822 days)** |  |  |  |  |  |
| PM_10_ (µg.m^-3^) | 9 | 17.9 | 9.4 | 5.0 | 78.1 |
| PM_ion_ (µg.m^-3^) | 101 | 5.5 | 4.5 | 0.4 | 45.7 |
| PM_rest_ (µg.m^-3^) | 110 | 12.4 | 8.8 | 0.0 | 70.2 |
| PM_2.5_ (µg.m^-3^) | 957 | 8.2 | 4.8 | 0.6 | 40.9 |
| Soot (µg.m^-3^) | 31 | 2.3 | 2.4 | 0.8 | 21.0 |

SD: Standard deviation

*PM_10_ and PM_rest_ available from 1990-2010 and PM_2.5_ available from 2006-2010

Warm period: April−September, cold period: October−March
